# A Dual-Gene Signature of PMAIP1 and GADD45A for Early Detection of Intrahepatic Cholangiocarcinoma in the Context of Primary Sclerosing Cholangitis

**DOI:** 10.3390/ijms27114826

**Published:** 2026-05-27

**Authors:** Bei Yao, Yiming Ma, Shuang Guan, Qiguang Zheng, Yanan Yu, Ran Jia, Yinli Shi, Zhiyong Hou, Zhong Wang, Jun Liu

**Affiliations:** Institute of Basic Research in Clinical Medicine, China Academy of Chinese Medical Sciences, Beijing 100700, China; 15191891270@163.com (B.Y.); mym6650362@163.com (Y.M.);

**Keywords:** primary sclerosing cholangitis, intrahepatic cholangiocarcinoma, single-cell RNA sequencing, malignant transformation, metabolic reprogramming

## Abstract

Primary sclerosing cholangitis (PSC) is a chronic inflammatory precursor associated with an increased risk of intrahepatic cholangiocarcinoma (ICC), yet identifying malignant features within the persistent inflammatory background remains challenging. In this study, a background-deviation framework was applied to explore malignant-associated determinants during PSC-associated cholangiocarcinogenesis. Single-cell RNA sequencing data from PSC, ICC tumor tissues, and adjacent non-tumor tissues were integrated, followed by functional enrichment, CellChat analysis, Monocle 2 pseudotime reconstruction, Non-negative Matrix Factorization (NMF), STRING/Cytoscape network analysis, and diagnostic signature construction using LASSO regression and exhaustive best subset selection. Single-cell profiling suggested disease-associated cellular remodeling, including cholangiocyte expansion in ICC samples. Functional and intercellular communication analyses indicated a putative transition from an immune-dominant PSC state toward a hyper-biosynthetic ICC-associated phenotype, accompanied by a possible MIF receptor-usage shift from CXCR4 to CD44. Monocle 2 and NMF further identified candidate malignant-associated trajectories and meta-programs, with MYC/TP63-related regulatory signals emerging as potential contributors. Based on these exploratory findings, best subset selection identified a two-gene transcriptomic candidate signature comprising PMAIP1 and GADD45A, which showed promising discriminative performance in internal cross-validation and an external tumor-versus-adjacent validation cohort. These findings provide a transcriptomic basis for further validation of PSC-associated cholangiocarcinogenesis and potential ICC surveillance markers.

## 1. Introduction

Intrahepatic cholangiocarcinoma (ICC) is a highly lethal malignancy arising from the biliary epithelium, characterized by rising global incidence and a 5-year survival rate below 20% [1,2]. Primary sclerosing cholangitis (PSC) is a chronic cholestatic liver disease defined by progressive biliary inflammation and fibrosis [3], serving as a major predisposing factor for ICC [4]. Clinical evidence indicates that the cumulative risk of hepatobiliary malignancy in patients with PSC reaches up to 20% over 30 years [5,6]. However, a critical clinical challenge remains: identifying early malignant transformation within a background of chronic inflammation is difficult, resulting in delayed diagnosis and poor prognosis.

Current understanding of the link between chronic inflammation and carcinogenesis posits that sustained pro-inflammatory signaling [7] creates a permissive microenvironment for tumor initiation [8]. However, the precise cellular evolutionary logic governing the transition from an inflammatory adaptive state to an overt malignant phenotype remains poorly defined. Previous genomic and transcriptomic studies have largely investigated PSC [9] and ICC [10] as separate entities, failing to comprehensively characterize the continuous dynamic changes that occur during disease progression. Crucially, it remains unclear whether malignancy arises as a de novo event replacing the inflammatory state, or if it evolves through the reprogramming of pre-existing inflammatory populations.

To address this issue, high-resolution mapping of cellular and transcriptional heterogeneity from inflammation-associated states to malignancy is needed. Single-cell RNA sequencing (scRNA-seq) [11] offers an opportunity to characterize cholangiocyte states and dissect transcriptional heterogeneity within complex tissue microenvironments. By integrating transcriptomic profiles across the PSC–ICC spectrum—comprising benign inflammatory tissues, paired adjacent non-tumor tissues, and established tumors—we aimed to define transcriptional patterns associated with inflammatory adaptation, peritumoral remodeling, and malignant progression. This strategy enabled us to distinguish “shared scaffolds” retained across disease states from “specific deviations” enriched in malignant-associated cholangiocyte programs. Here, the shared scaffold refers to conserved inflammatory and stress-response programs that are present across PSC, ICC-adjacent tissue, and ICC tumor tissue, whereas specific deviations denote malignant-enriched transcriptional, metabolic, or microenvironmental features that emerge beyond this common inflammatory background.

**Figure 1:**
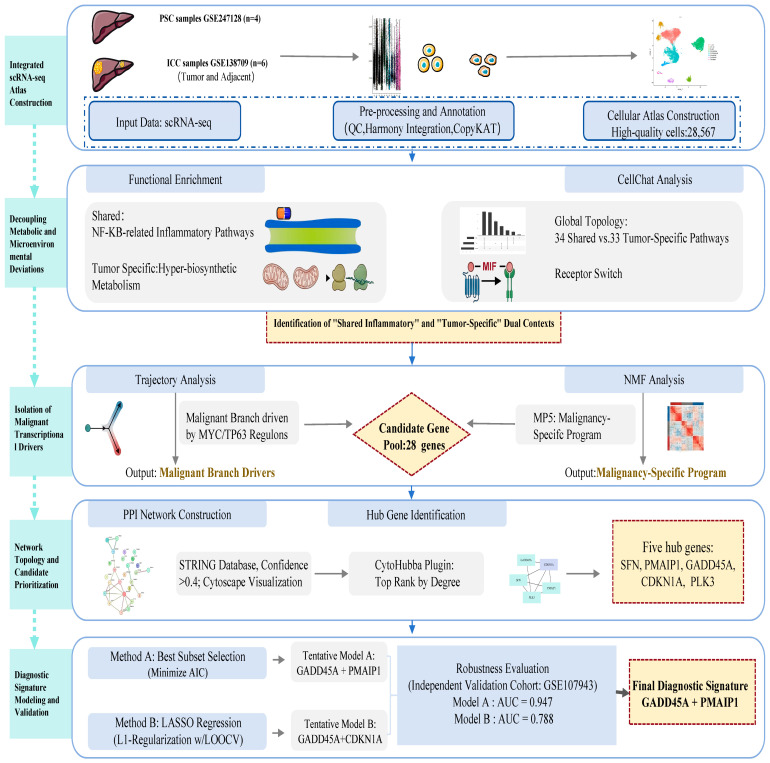
Schematic workflow for identifying malignant progression determinants and diagnostic signatures in PSC-associated cholangiocarcinogenesis.

In this study, we performed an integrated single-cell transcriptomic analysis of PSC-associated cholangiocarcinogenesis and proposed a background-deviation framework. Rather than supporting a simple linear model in which inflammation is replaced by malignancy, our findings suggest that malignant-associated cholangiocyte states may emerge through the superimposition of hyper-biosynthetic and oncogenic stress programs upon a persistent inflammatory scaffold. Building on this framework, we identified PMAIP1 and GADD45A as a candidate dual-gene transcriptomic signature that captures malignant-associated deviation from the inflammatory background. This work provides a conceptual and molecular basis for further investigation of PSC-associated cholangiocarcinogenesis. The overall workflow for identifying malignant progression determinants and diagnostic signatures is summarized in Figure 1.

## 2. Results

### 2.1. Comparative Profiling of the Cellular Landscape Across the PSC–ICC Spectrum Through Single-Cell Analysis

To elucidate the cellular transitions associated with PSC-associated cholangiocarcinogenesis, an integrated single-cell RNA sequencing (scRNA-seq) dataset was constructed (Figure S1, Section 4). This dataset incorporated data from primary sclerosing cholangitis (PSC; *n* = 4) and intrahepatic cholangiocarcinoma (ICC; *n* = 3 tumors with *n* = 3 matched adjacent non-tumor tissues), capturing the inflammatory, peritumoral, and malignant states across the disease spectrum.

**Figure 2:**
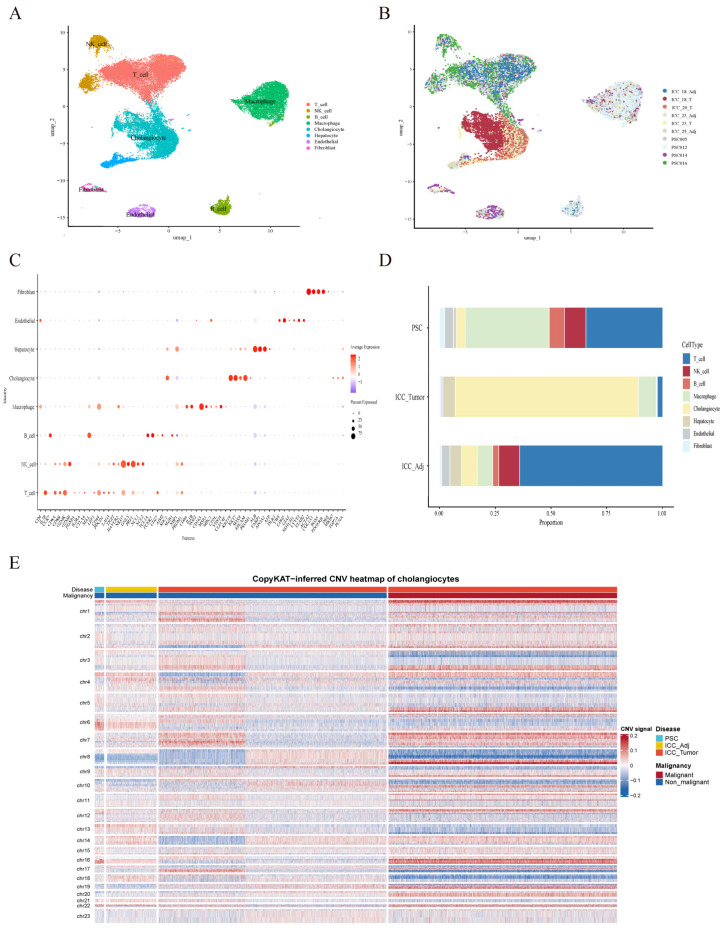
Single-cell transcriptomic landscape of PSC and ICC tissues. (**A**) UMAP visualization of all sequenced cells, color-coded by identified cell lineages. (**B**) UMAP plot of all cells, color-coded by sample origins. (**C**) Dot plot showing the expression of canonical marker genes used to define each cell type. The color intensity represents the average expression level, and the dot size indicates the percentage of cells expressing the marker. (**D**) Bar plot illustrating the relative proportions of each cell type across three disease states: PSC, ICC-Adj, and ICC-Tumor. (**E**) CopyKAT-inferred CNV heatmap of cholangiocytes. Columns represent individual cholangiocytes and rows represent genomic bins ordered by chromosomal position. Top annotations indicate disease group and CopyKAT-based binary malignancy status.

Cell types were identified based on canonical marker genes and the CellMarker database [12]. We visualized the resulting cellular landscape using Uniform Manifold Approximation and Projection (UMAP), defining eight major lineages: T cells, B cells, NK cells, macrophages, cholangiocytes, hepatocytes, endothelial cells, and fibroblasts (Figure 2A–C).

Cell-type composition showed disease-associated differences across PSC, ICC-adjacent tissue (ICC-Adj), and ICC tumor tissue (ICC-Tumor) (Figure 2D). PSC samples displayed an immune-enriched architecture, characterized by relatively higher proportions of macrophages and lymphoid populations, including T/NK cells and B cells. In contrast, cholangiocytes were more abundant in ICC-Tumor samples, whereas ICC-Adj samples showed a prominent T-cell component. Across the three tissue states, cholangiocyte abundance appeared to increase from PSC to ICC-Tumor, while macrophage abundance showed a decreasing pattern, and fibroblast proportions remained comparatively stable (Figure 2D and Figure S2).

Given that cholangiocytes are widely regarded as the putative cell of origin for ICC [13], their relative enrichment in ICC-Tumor samples provided a cellular basis for subsequent cholangiocyte-focused analyses. To further distinguish malignant-like cholangiocytes, CopyKAT-based CNV inference was applied to the cholangiocyte population. The resulting CNV heatmap showed broad CNV-like alterations in CopyKAT-defined malignant cholangiocytes, supporting their malignant-associated aneuploid annotation for downstream analyses (Figure 2E). Collectively, these dataset-level observations provided an initial single-cell context for investigating epithelial and microenvironmental remodeling across the PSC–ICC spectrum.

### 2.2. Functional Divergence and Metabolic Reprogramming of Cholangiocytes During Malignant Progression

**Figure 3:**
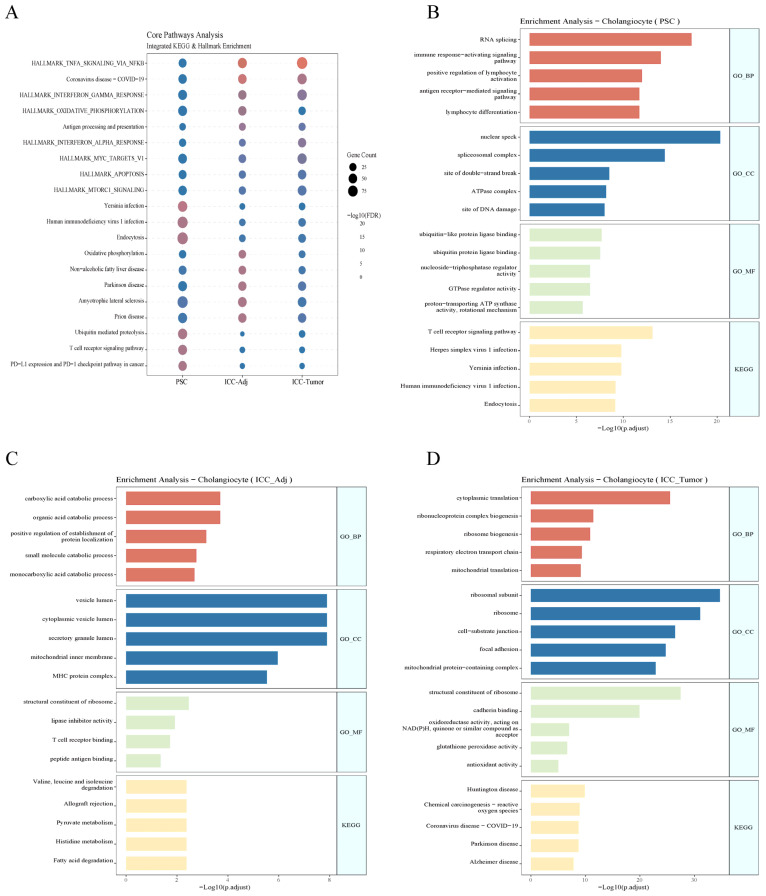
Functional landscape and stage-specific divergence of cholangiocytes during PSC-associated cholangiocarcinogenesis. (**A**) Bubble plot illustrating the integrated enrichment of Hallmark and KEGG pathways across PSC, ICC-Adj, and ICC-Tumor states. Conserved inflammatory pathways, particularly the NF-κB-signaling pathway (TNFA_SIGNALING_VIA_NFKB), are identified as a persistent “inflammatory scaffold” across all disease stages. The dot size represents the gene count, and the color intensity indicates the significance level. (**B**–**D**) Functional enrichment analysis showing the specific GO (Biological Process, Cellular Component, and Molecular Function) and KEGG terms for cholangiocytes in PSC (**B**), ICC-Adj (**C**), and ICC-Tumor (**D**) tissues.

Following the characterization of distinct cellular shifts across disease states, further investigation focused on the functional heterogeneity of cholangiocytes in PSC, ICC-adjacent, and ICC-Tumor tissues. Enrichment analysis was performed based on differentially expressed genes (DEGs) identified across these conditions (Tables S1 and S2; Figures S3–S6). Functional enrichment analysis suggested that NF-κB-related inflammatory programs, particularly HALLMARK_TNFA_SIGNALING_VIA_NFKB, were commonly represented across PSC [14] and ICC samples (Tables S3–S6; Figure S7). This finding indicates that pro-inflammatory signaling may persist as a shared inflammatory scaffold throughout the PSC–ICC spectrum. Consequently, inflammation-related transcriptional activity alone may be insufficient to distinguish malignancy-associated epithelial states from the benign inflammatory background (Figure 3A).

Despite this shared inflammatory background, cholangiocytes from different tissue states displayed distinct functional enrichment patterns (Figure 3B–D). In the PSC stage, cholangiocyte function was primarily associated with immune response interactions, characterized by the enrichment of “T cell receptor signaling” and “antigen processing and presentation”. This reflects a reactive state in which biliary epithelial cells actively participate in mucosal immunity. In stark contrast, ICC-Tumor cholangiocytes exhibited a fundamental transition toward a hyper-biosynthetic and bioenergetic phenotype. The malignant state was defined by the marked upregulation of ribosome biogenesis (e.g., “rRNA processing,” “ribosome large subunit assembly”) and oxidative phosphorylation. To provide quantitative support for this observation, module scores for predefined representative gene programs related to biosynthesis, energy metabolism, and oncogenic transcriptional regulation were calculated in cholangiocytes. These scores increased stepwise from PSC to ICC-adjacent and ICC-tumor cholangiocytes, supporting a malignant-associated hyper-biosynthetic and bioenergetic transcriptional state (Table S7). This enrichment of protein synthesis machinery and mitochondrial respiratory pathways suggests that the core feature of malignant transformation is driven by specific metabolic rewiring rather than a mere continuation of inflammation. Additionally, cholangiocytes in ICC-adjacent tissues displayed an intermediate “metabolic adaptation” phenotype, characterized by the enrichment of fatty acid degradation (Table S2).

Collectively, these findings support the background-deviation framework: PSC and ICC share a generalized inflammatory scaffold, whereas ICC-Tumor cholangiocytes exhibit additional malignant-associated biosynthetic and metabolic features superimposed upon this background.

### 2.3. Remodeling of Cholangiocyte-Centered Intercellular Communication Networks

**Figure 4:**
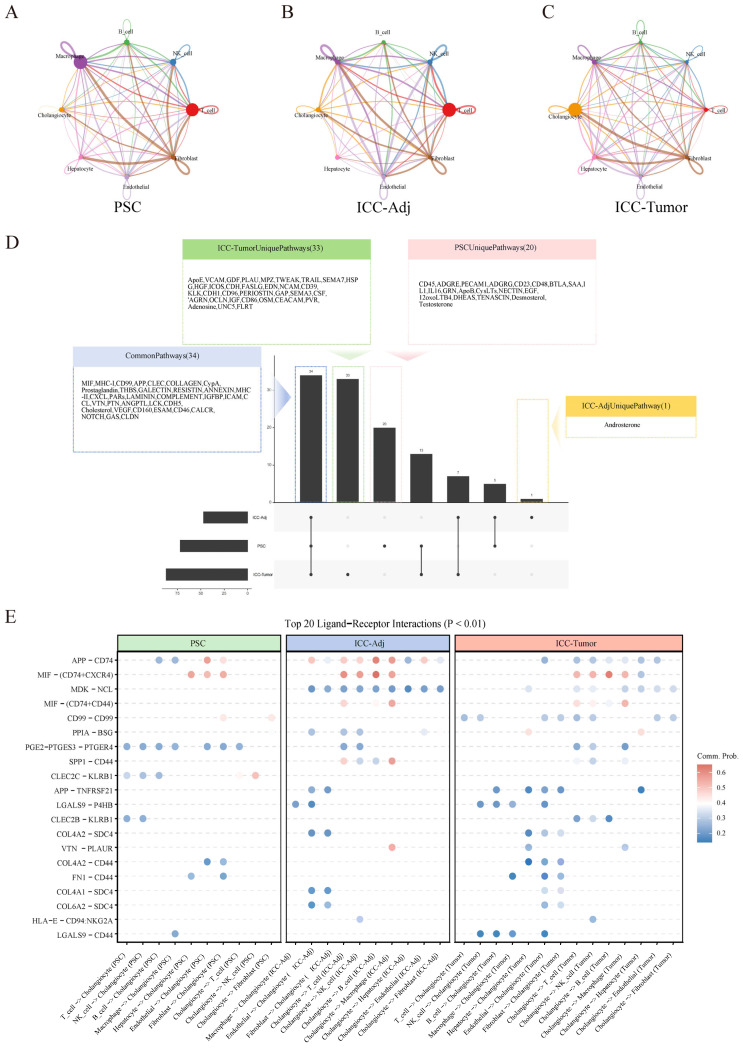
Alterations in intercellular communication networks and ligand-receptor interactions during PSC-associated cholangiocarcinogenesis. (**A**–**C**) Global cell–cell communication networks in PSC (**A**), ICC-Adj (**B**), and ICC-Tumor (**C**) tissues. The lines indicate interactions between cell types, where line thickness represents the interaction strength and number of ligand-receptor pairs. (**D**) Upset plot showing the shared and stage-specific signaling pathways among the three disease states. The intersection highlights the conserved signaling core, while the unique sets represent functional shifts specific to malignant progression. (**E**) Dot plot illustrating the ligand-receptor pairs of cholangiocytes acting as either senders or receivers across the three disease states. The dot size represents the communication probability, and the color intensity indicates the computed *p*-value.

While functional profiling delineates the intrinsic metabolic reprogramming characteristic of the malignant state, these cellular adaptations are inevitably shaped by extrinsic microenvironmental signals. To delineate putative extrinsic microenvironmental changes across PSC, ICC-Adj, and ICC-Tumor states, intercellular communication patterns were inferred using CellChat (Figure 4A–C). The analysis suggested a topological reconfiguration of the cellular communication network. In the PSC stage, the inferred network was mainly centered on interactions involving macrophages and fibroblasts. In ICC-Tumor, fibroblasts and cholangiocytes emerged as prominent communication hubs, suggesting enhanced epithelial–stromal communication in the malignant tissue context (Figures S8–S10).

Integration of global pathway-level analysis with ligand-receptor (L-R) profiling suggested two layers of microenvironmental remodeling (Tables S8–S10; Figure S11). First, a conserved signaling foundation was observed across the disease spectrum. A total of 34 signaling pathways, including MIF, Prostaglandin, and VEGF, were shared among PSC, adjacent, and tumor tissues (Figure 4D, Figures S12 and S13). At the ligand-receptor level, inflammatory interactions such as PGE2-PTGER4 and MIF-(CD74+CXCR4) were broadly detected across tissue states. These findings are consistent with the presence of a shared inflammatory scaffold (Figure 4D).

Superimposed on this shared background, ICC-Tumor tissues showed enrichment of tumor-associated pathways and a potential “receptor switch” pattern (Figure S14). Analysis identified 33 pathways preferentially enriched in the ICC tumor microenvironment, including invasive or tumor-associated modules such as ApoE, HGF, and TRAIL. This pattern was further accompanied by preferential detection of SPP1-CD44- and MDK-NCL-signaling axes in tumor and adjacent tissues, while PSC-associated immune pathways, such as IL1 and CD45, were relatively attenuated.

Notably, a shift in cellular response to conserved MIF signaling was observed. Although the MIF ligand was broadly present, its inferred receptor usage appeared to shift from the inflammatory background-associated MIF-(CD74+CXCR4) axis toward the ICC-associated MIF-(CD74+CD44) axis. The detection of MIF-(CD74+CD44) signaling in adjacent non-tumor tissues may be consistent with field-like peritumoral remodeling [15,16] in line with the concept that tumor-adjacent tissues can harbor molecular alterations distinct from truly healthy tissues. However, this pattern may also reflect tumor-adjacent inflammatory or stromal responses, differences in tissue sampling, cell-state composition, or dataset-related batch effects between PSC and ICC cohorts. In summary, both metabolic and microenvironmental profiles support a “Background-Deviation” pattern, in which malignant-associated features are superimposed upon a conserved inflammatory scaffold through altered ligand-receptor usage.

### 2.4. Identification of Transcriptional Drivers and Regulatory Networks Governing Malignant Lineage Specification

**Figure 5:**
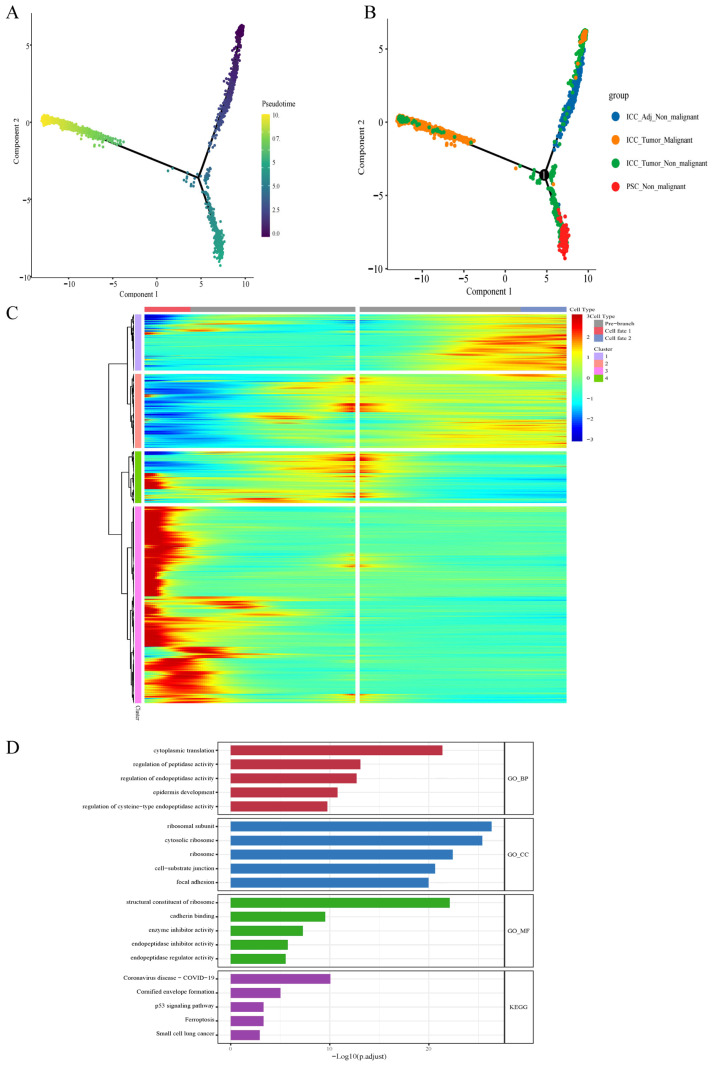
Pseudotime trajectory and branch-specific functional determinants of cholangiocyte malignant progression. (**A**) Monocle 2 pseudotime trajectory of all cholangiocytes, with cells ordered according to their developmental progress. The color gradient represents the evolution of pseudotime. (**B**) Distribution of cholangiocytes from different sample groups along the pseudotime trajectory. Individual cells are color-coded by their identified cell types within each disease state. (**C**) BEAM (Branched Expression Analysis Modeling) heatmap displaying the kinetic changes in gene expression at the branching point. Fate 1 represents the inflammatory non-malignant lineage, while Fate 2 signifies the malignant cholangiocyte lineage. (**D**) GO and KEGG enrichment analysis of genes associated with the transition toward the malignant lineage (Fate 2). The results highlight the functional drivers of malignant progression.

Following the macroscopic delineation of specific malignant features and the shared inflammatory background, the dynamic transcriptional logic governing lineage specification was investigated. To identify the molecular drivers regulating the transition from a shared inflammatory state to a malignant fate, the developmental trajectory of cholangiocytes was reconstructed using Monocle 2 (Figures S15–S21). The analysis revealed that all cholangiocytes originate from a common transcriptional root (Figure 5A,B), regardless of their ultimate fate. This root is characterized by a “shared stress response,” defined by the transient induction of immediate-early genes (IEGs) [17], including JUN, CYR61, and CXCL family members. As cells progressed along the trajectory, this initial stress signature diminished, while lineage-specific markers, such as S100 family members and the antioxidant regulator PRDX1, gradually accumulated (Figures S22 and S23).

The trajectory bifurcated into two distinct termini: an “inflammatory adaptation branch,” predominantly populated by PSC cells, and a “malignant progression branch,” primarily composed of ICC-Tumor cells. To isolate the drivers directing this bifurcation, Branched Expression Analysis Modeling (BEAM) was applied (Figure 5C; Table S11). A gene module specifically activated along the malignant branch was identified, showing significant enrichment in ribosome biogenesis, oxidative phosphorylation, and cell cycle progression (Figure 5D). This provides a mechanistic link to the hyper-biosynthetic phenotype observed earlier, confirming the metabolic switch as an intrinsic developmental process rather than a passive byproduct.

Screening for upstream regulators within this module highlighted MYC- and TP63-associated regulatory signals as candidate contributors to the malignant-associated branch (Table S12). MYC [18] serves as a critical node linking extrinsic signals to internal metabolic reprogramming. Meanwhile, the induction of TP63, [19] along with stemness factors (KLF4) [20] and EMT regulators (SNAI2), suggests that malignant cholangiocytes acquire a dedifferentiated state to survive microenvironmental pressure. These trajectory-driving genes represent specific “markers” that distinguish malignancy from the inflammatory background, providing a high-confidence candidate pool for the construction of the diagnostic signature.

### 2.5. NMF Meta-Program Analysis Decouples Stable and Specific Malignant Transcriptional Programs

While pseudotime analysis reconstructed the dynamic evolutionary trajectory of malignancy, mathematical deconvolution was required to isolate stable, tumor-specific signals from the pervasive inflammatory background. To define fixed transcriptional meta-programs (MPs) characterizing each disease state and to differentiate malignant features from shared signals, Non-negative Matrix Factorization (NMF) was employed.

**Figure 6:**
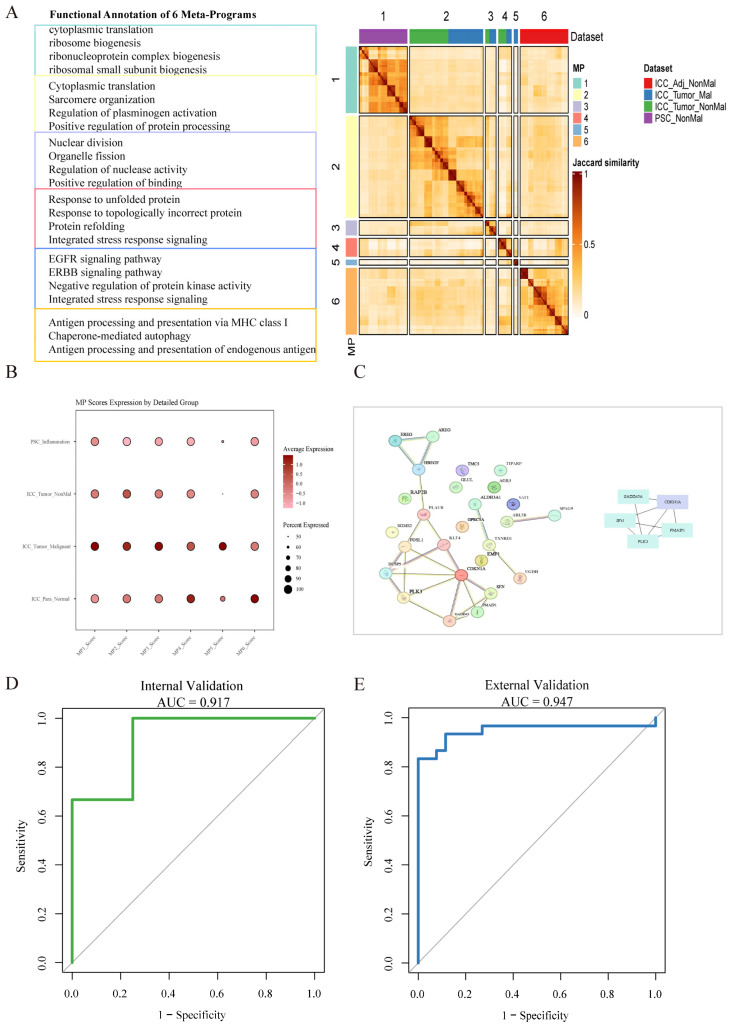
Identification of malignant meta-programs and construction of the diagnostic signature for PSC-associated cholangiocarcinogenesis. (**A**) Non-negative Matrix Factorization (NMF) analysis identifies six distinct meta-programs (MPs) and their corresponding functional enrichment profiles. The heatmap displays the relative expression of top-ranked genes for each MP, with associated biological functions annotated alongside. (**B**) Relative expression levels of the six identified MPs across four defined groups: PSC non-malignant, ICC-Adj non-malignant, ICC-Tumor non-malignant, and ICC-Tumor malignant cholangiocytes (as identified by CopyKAT). (**C**) Protein-Protein Interaction (PPI) network of the top-ranked genes from malignant-specific MPs, visualized via STRING and Cytoscape. The five central hubs (hub genes) were prioritized based on degree centrality. The five highest-degree hub genes were CDKN1A (degree = 7), GADD45A (degree = 4), PLK3 (degree = 4), PMAIP1 (degree = 4), and SFN (degree = 4). (**D**,**E**) Performance and validation of the dual-gene diagnostic signature (PMAIP1 and GADD45A) derived from the best subset selection method. ROC curves illustrate the diagnostic accuracy (AUC) in both the discovery (internal) cohort (**D**) and the independent validation (external) cohort (**E**).

Six recurrent meta-programs were identified in the present dataset (Figure 6A; Tables S13–S18). Intriguingly, peritumoral cholangiocytes (derived from ICC-Adj) were not transcriptionally quiescent but exhibited a distinct stress-adaptive phenotype, predominantly characterized by MP4 and MP6. MP4 was enriched in the “unfolded protein response (UPR)” and “protein refolding,” suggesting that adjacent cells activate specific adaptation mechanisms to survive the perturbed peritumoral microenvironment. MP6 was defined by the high expression of MHC-I antigen presentation genes, indicating that these cells maintain immune visibility, which contrasts with the immune evasion typical of established tumors. These findings are consistent with the “shared stress response” identified in the pseudotime analysis, suggesting that adjacent tissues represent an intermediate state where cells experience environmental stress prior to full malignant reprogramming (Figure 6B and Figure S24).

Crucially, MP5 was identified as a malignancy-specific program, distinct from the inflammatory programs observed in PSC (Figure 6B and Figure S25). Functionally, MP5 was enriched in EGFR/ERBB signaling and the negative regulation of protein kinase activity. This meta-program represents the specific oncogenic module superimposed upon the background inflammatory stress. Additionally, MP3, enriched in nuclear division, was co-activated in ICC-Tumor, further supporting the proliferative capacity driven by the MYC regulatory network. Thus, MP5 provided an additional layer of support for the malignant-associated transcriptional patterns suggested by pseudotime analysis, although its stability requires further validation in larger datasets.

### 2.6. Integrative Identification and Validation of a Malignant-Specific Diagnostic Signature Within the PSC-ICC Spectrum

To bridge the gap between high-dimensional single-cell data and clinical applicability, an integrative approach was employed to develop a robust diagnostic signature capable of distinguishing early ICC from the pervasive inflammatory background of PSC. This strategy intersected lineage-driving genes identified from pseudotime analysis with the malignant-specific meta-program (MP5) derived from NMF deconvolution, resulting in a high-confidence pool of 28 candidate genes (Table S19). To ensure the statistical significance and biological relevance of these candidates, a Protein–Protein Interaction (PPI) network was constructed using STRING (v12.5) and Cytoscape (v3.10.4). The top five hub genes—SFN, PMAIP1, GADD45A, CDKN1A, and PLK3—were prioritized for downstream diagnostic modeling based on degree centrality (Figure 6C; Table S20).

Subsequently, we applied an exhaustive “best subset selection” method based on multivariate logistic regression to optimize feature selection (Table S20; Figure S26). This approach identified a two-gene candidate transcriptomic signature comprising PMAIP1 and GADD45A. By leveraging the synergistic signaling of stress-response and pro-apoptotic regulators, the signature effectively captures the malignant “deviations” superimposed on the inflammatory baseline. These genes undergo specific transcriptional reconfiguration during PSC-associated cholangiocarcinogenesis, reflecting core oncogenic mechanisms. In the discovery cohort, internal leave-one-out cross-validation (LOOCV) yielded an AUC of 0.917 (Figure 6D), suggesting promising discriminative performance within the available dataset while indicating that the model performance should be interpreted in the context of the discovery-cohort design. The PMAIP1/GADD45A signature was further evaluated in the independent external cohort GSE107943. In this dataset, the control background comprised adjacent non-tumor tissues rather than inflammatory PSC samples; therefore, this analysis was interpreted as a tumor-versus-adjacent validation rather than direct validation in a PSC surveillance setting. Within this context, the signature showed high discriminative performance, with an AUC of 0.947 (Figure 6E). This result supports preliminary cross-dataset tumor-discriminative potential.

To ensure model robustness and benchmark against standard algorithms, we compared the performance of a LASSO logistic regression model (Figure S27). Although the LASSO method selected GADD45A and CDKN1A as predictors, this combination resulted in a lower AUC of 0.788 in the external validation dataset (Figure S28). The superior performance of the best subset selection method underscores the unique diagnostic value of the PMAIP1/GADD45A combination. This signature may reflect a biologically plausible stress-superimposition pattern associated with malignancy. Nevertheless, its generalizability and potential utility for monitoring malignant progression in high-risk PSC patients require further evaluation in independent PSC-specific and longitudinal cohorts.

## 3. Discussion

This study delineates the transcriptional landscape underlying the PSC-ICC spectrum and provides support for the “background-deviation” framework. Malignant-associated progression was characterized by a phenotypic shift toward hyperbiosynthesis and microenvironmental remodeling involving the MIF-CD44 axis. Trajectory analysis highlighted MYC/TP63-related regulatory signals potentially associated with this deviation, while NMF helped decouple malignant-associated transcriptional programs from pervasive inflammatory signals. Protein network analysis further refined the biological connectivity of candidate drivers and prioritized hub genes for downstream modeling. This integrative workflow ultimately identified PMAIP1 and GADD45A as a candidate transcriptomic signature that characterizes malignancy as a stress-superimposition state rather than a mere escalation of inflammation.

The existing literature supports the critical role of PMAIP1 [21] and GADD45A in stress responses and validates their capacity to distinguish malignancy. PMAIP1 encodes NOXA, a BH3-only pro-apoptotic protein [22], and its transcriptional upregulation may serve as an indicator of metabolic and hypoxic stress. Our functional analysis revealed a hyper-biosynthetic phenotype in ICC, a state inevitably accompanied by the Unfolded Protein Response (UPR) and hypoxia. Consistent with this, studies indicate that PMAIP1 transcription is rigorously induced by endoplasmic reticulum (ER) stress and hypoxia [23]. Although malignant cells may attenuate PMAIP1 protein levels via the SAG-UPS system [24] to evade apoptosis, the elevated PMAIP1 transcript levels observed here may still reflect persistent “oncogenic stress” at the mRNA level. Thus, PMAIP1 should be interpreted in this study as a transcriptomic stress indicator that helps distinguish metabolically burdened malignant-associated cells from the inflammatory background.

Complementing this, GADD45A functions as a specific monitor for genomic instability [25]. Our trajectory analysis identified the MYC/TP63 network as the driver of the malignant branch, and MYC-driven proliferation [26] is a well-established inducer of replication stress. The sharp upregulation of GADD45A serves as a direct cellular response to this replication pressure [27] and DNA damage [28]. Unlike the chronic, low-level damage response observed in benign PSC, the elevation of GADD45A in the signature may reflect genomic stress associated with malignant transformation. In summary, the PMAIP1/GADD45A signature characterizes a transcriptomic pattern combining metabolic and genomic stress, thereby helping distinguish malignant-associated deviation from the inflammatory background.

Beyond its direct diagnostic utility, the identification of the PMAIP1/GADD45A signature offers a conceptual refinement to the current understanding of PSC-associated carcinogenesis. Traditionally, clinical surveillance of PSC has relied on detecting cellular proliferation or structural dysplasia [29,30]. However, within a chronic inflammatory context, benign reactive biliary lesions frequently exhibit high proliferative indices, limiting the specificity of such markers [31]. The synergistic upregulation of PMAIP1 and GADD45A indicates that malignant cholangiocytes operate under intense metabolic and genomic stress, which is distinct from the homeostatic stress of benign inflammation. Consequently, this study proposes a shift in early detection strategies: moving from monitoring non-specific inflammatory progression to targeting the specific molecular footprints of oncogenic stress.

Importantly, this stress-based signature also provides a clinically interpretable link to therapeutic vulnerabilities associated with malignant stress adaptation. PMAIP1/NOXA places this marker within the mitochondrial apoptosis-regulatory network, and this connection is translationally relevant because BH3-mimetic strategies have already shown activity in cholangiocarcinoma-related models. For example, obatoclax, a pan-BCL-2 family antagonist with activity against MCL1, induced Bax activation and apoptosis in cholangiocarcinoma cells and produced antitumor effects in an orthotopic cholangiocarcinoma model [32]. In addition, recent evidence in intrahepatic cholangiocarcinoma showed that inhibition of the stress-response regulator HSF1 could be potentiated by the Bcl-xL/Bcl-2/Bcl-w inhibitor ABT-263 (navitoclax), further supporting the therapeutic relevance of apoptosis-threshold modulation in this disease context [33]. In parallel, GADD45A is closely linked to DNA-damage and replication-stress responses, connecting this signature to DNA damage response-directed treatment strategies. Consistently, homologous recombination deficiency has been reported in a subset of biliary tract cancers, supporting the potential use of PARP inhibitors and DNA-damaging regimens [34]. Moreover, combined PARP/ATR inhibition with olaparib and ceralasertib/AZD6738 has shown antitumor activity in biliary tract cancer models [35]. Together, these findings suggest that the PMAIP1/GADD45A signature not only distinguishes malignant-associated stress from the chronic inflammatory background, but it also highlights apoptosis-threshold and DNA-damage-response pathways as clinically relevant axes for therapeutic exploration in PSC-associated cholangiocarcinogenesis.

Our study has several limitations. First, the integrated scRNA-seq analysis was based on four PSC samples, three ICC tumor samples, and three adjacent non-tumor samples. Although this dataset enabled an exploratory comparison across inflammatory, peritumoral, and malignant tissue states, the observed differences in cell-type composition, pseudotime trajectories, NMF-derived meta-programs, and CellChat-inferred interactions may be influenced by inter-individual heterogeneity, tissue sampling variation, dataset-related technical effects, and incompletely available clinical covariates. In particular, the curated metadata revealed an imbalanced sex distribution, with all PSC samples from GSE247128 derived from male patients and the ICC samples from GSE138709 derived from both female and male patients (Table S22). Moreover, age, PSC disease duration, and detailed medication history were incompletely available. Given the limited patient-level sample size and incomplete covariate annotation, formal adjustment for these potential confounders was not statistically robust. Therefore, these single-cell findings should be interpreted as exploratory patterns rather than definitive causal evidence of PSC-to-ICC progression. Second, the external validation cohort consisted of ICC tumor and adjacent non-tumor tissues rather than PSC inflammatory tissues. Thus, the external AUC should be viewed as preliminary evidence of tumor-discriminative potential across datasets, but not as a direct validation of clinical surveillance performance in PSC patients. Third, the PMAIP1/GADD45A signature was constructed from transcriptomic data, and protein-level validation by immunohistochemistry or functional assays remains necessary before clinical translation. In conclusion, this study proposes a background-deviation framework in which malignant-associated stress programs are superimposed upon a persistent inflammatory scaffold. The PMAIP1/GADD45A signature represents a candidate transcriptomic feature linked to this model, providing a molecular basis for further validation of PSC-associated cholangiocarcinogenesis.

## 4. Materials and Methods

### 4.1. Data Acquisition and Integration

Transcriptomic datasets were retrieved from the Gene Expression Omnibus (GEO; National Center for Biotechnology Information, U.S. National Library of Medicine, Bethesda, MD, USA) (https://www.ncbi.nlm.nih.gov/geo/, accessed on 6 March 2026). To profile the cellular landscape of PSC-associated cholangiocarcinogenesis, scRNA-seq data from primary sclerosing cholangitis (PSC; GSE247128) and intrahepatic cholangiocarcinoma (ICC; GSE138709) were integrated, yielding 10 samples encompassing PSC inflammatory tissues (*n* = 4), ICC-Tumor tissues (*n* = 3), and adjacent non-tumor tissues of intrahepatic cholangiocarcinoma (ICC-Adj; *n* = 3) (Table S23). Datasets were included if they were derived from human liver or biliary tissues, had clearly annotated disease or tissue origins, and provided expression matrices suitable for downstream analysis. Samples lacking clear tissue annotation, disease-state information, or usable expression data were excluded. Available patient- and sample-level demographic and clinical metadata for these GEO scRNA-seq datasets were manually curated from GEO SOFT files, BioSample/SRA records, and the Supplementary Materials of the original publications, and they are summarized in Table S21. The curated variables included sample identity, disease group, tissue type, sex, age, disease duration, medication or treatment status, and other available clinical or pathological characteristics. Variables not reported in the public GEO/SRA metadata or the corresponding Supplementary Materials were marked as “not available”. For external validation, an independent bulk RNA-seq cohort (GSE107943; 57 samples, including 30 ICC tumor samples and 27 adjacent non-tumor samples) comprising paired ICC tumor and adjacent tissues was analyzed. Data processing was conducted in R software (v4.4.1; R Foundation for Statistical Computing, Vienna, Austria) using Seurat (v5.3.0; Satija Lab, New York Genome Center, New York, NY, USA). Quality control was performed by excluding cells with fewer than 300 or more than 4500 detected genes, or with more than 15% mitochondrial counts [36].

The 15% mitochondrial threshold was selected as a permissive cutoff to avoid excessive depletion of hepatobiliary epithelial cells under inflammatory and tumor-associated stress conditions. In PSC and ICC tissues, cholangiocytes may exhibit increased mitochondrial transcription associated with metabolic stress, oxidative phosphorylation, hypoxia-related responses, and tissue dissociation-associated stress. Therefore, an overly stringent mitochondrial threshold may remove biologically relevant stress-associated cholangiocytes.

To evaluate whether the mitochondrial threshold influenced the key marker-level findings, a focused sensitivity analysis was further performed using a stricter 10% mitochondrial cutoff. Specifically, the annotated single-cell object was subsetted to retain only cells with mitochondrial gene percentages ≤ 10%. Cell retention, cholangiocyte distribution across disease groups, CopyKAT-based malignant/non-malignant cholangiocyte composition, and PMAIP1/GADD45A expression patterns were then compared between the original 15% object and the strict 10% subset. A simple dual-gene expression score, calculated as the mean normalized expression of PMAIP1 and GADD45A at the single-cell level, was used only for this sensitivity analysis. The corresponding mitochondrial-threshold assessment and focused 10% cutoff sensitivity results are provided in Figure S29 and Table S24. After log-normalization, doublets were identified and removed using DoubletFinder (V.2.0.6; University of California, San Francisco, San Francisco, CA, USA) [37].

Cell-cycle scores were calculated based on S- and G2/M-phase markers and regressed out during scaling to mitigate proliferation-associated variation.

### 4.2. Batch Correction and Cell Type Annotation

Batch effects across datasets were corrected using Harmony (v1.2.3; Broad Institute, Cambridge, MA, USA) [38] on the top 30 principal components derived from 3000 highly variable genes [39]. Dimensionality reduction was performed with Uniform Manifold Approximation and Projection [40] (UMAP), and clustering was conducted using Seurat functions FindNeighbors and FindClusters (resolution = 0.3). Cell types were annotated based on established marker genes with reference to the CellMarker database (CellMarker 2.0; College of Bioinformatics Science and Technology, Harbin Medical University, Harbin, Heilongjiang, China). [12].

### 4.3. Identification of Malignant Cells

Malignant cholangiocytes were identified using CopyKAT (v1.1.0; Navin Lab, University of Texas MD Anderson Cancer Center, Houston, TX, USA) [41], which infers large-scale copy number variation (CNV) profiles from scRNA-seq data via Bayesian segmentation. Cholangiocytes were classified into aneuploid and diploid categories according to inferred CNV patterns [42] (Figure S30). In the primary binary classification used for downstream analyses, aneuploid cholangiocytes were defined as malignant, whereas non-aneuploid cholangiocytes were treated as non-malignant. Cells with available CopyKAT CNA profiles were used to generate the CNV heatmap (Figure 2E). CopyKAT-derived CNV burden was calculated from the inferred CNA matrix as a quantitative measure of CNV deviation, and sensitivity analyses were performed by evaluating PMAIP1/GADD45A expression across CNV-burden-based threshold settings (Figure S31; Tables S25–S27).

InferCNV (V.1.27.0; Broad Institute of MIT and Harvard, Cambridge, MA, USA) was further used as an orthogonal CNV inference approach. Non-tumor cholangiocytes from PSC inflammatory tissues, adjacent non-tumor tissues, and ICC-Tumor samples were selected as lineage-matched reference cells, while ICC-tumor cholangiocytes were treated as observation cells. InferCNV-derived gene-level CNV burden was calculated as the mean absolute deviation of the final InferCNV expression matrix from the neutral baseline (Figure S32; Table S28).

### 4.4. Pseudotime Trajectory and Transcriptional Regulation Analysis

Pseudotime trajectory analysis was performed using Monocle 2 (v2.10.0; Trapnell Lab, University of Washington, Seattle, WA, USA) [43] to reconstruct the evolutionary lineage of cholangiocytes. The DDRTree algorithm was used for dimensionality reduction and cell ordering. To identify key molecular drivers of lineage fate (inflammatory vs. malignant), Branched Expression Analysis Modeling (BEAM) [43] was applied. Genes with significant branch-dependent expression patterns (*q*-value < 1 × 10^−4^) were identified.

To uncover the regulatory network driving the malignant trajectory, a list of human transcription factors (TFs) [44] was retrieved from the org.Hs.eg.db annotation package (v3.22.0; Bioconductor, Buffalo, NY, USA) using the AnnotationDbi package (v1.66.0; Bioconductor, Buffalo, NY, USA). The select function was used to query genes associated with the Gene Ontology term “DNA-binding transcription factor activity” (GO:0003700) [44]. These TFs were then intersected with the BEAM-significant genes to identify key regulatory drivers.

### 4.5. Non-Negative Matrix Factorization (NMF) Analysis

To decouple stable malignant transcriptional programs from the inflammatory background, Non-negative Matrix Factorization (NMF) [45] was performed using the NMF R package (v0.28; CRAN, R Foundation for Statistical Computing, Vienna, Austria). The analysis was conducted independently on four subgroups (PSC non-malignant, ICC-Tumor malignant, ICC-Tumor non-malignant, and ICC-adjacent non-malignant cholangiocytes) using the “brunet” algorithm. Factorization rank (*k*) was tested from 4 to 9, with 30 iterations (*nrun* = 30) for each *k*. To assess the stability of NMF decomposition across the tested ranks, consensus-based diagnostic metrics were calculated, including the cophenetic correlation coefficient, dispersion, reconstruction residuals, silhouette width, silhouette consensus, and sparseness of the W and H matrices. The corresponding rank-stability diagnostic plots and numerical metrics are provided in Figure S33 and Table S29, respectively. Robust programs were defined based on recurrent top-gene overlap across different ranks within the same subgroup using Jaccard similarity, and meta-programs (MPs) [46] were identified by hierarchical clustering of these robust programs based on Jaccard similarity. The robust-program clustering structure was further evaluated using hierarchical clustering and within- versus between-meta-program Jaccard similarity analysis (Figure S33). The top 50 genes with the highest feature scores in each MP were defined as the core gene signatures.

### 4.6. Functional Enrichment and Cell-Cell Communication

Biological functions of differentially expressed genes (DEGs) and NMF meta-programs were annotated using Over-Representation Analysis (ORA) via the clusterProfiler package (v4.8.1; Bioconductor, Buffalo, NY, USA) [47]. Gene sets were retrieved from Gene Ontology (GO) [48], Kyoto Encyclopedia of Genes and Genomes (KEGG) [49], and MSigDB Hallmark collections [50]. Intercellular communication networks were inferred using CellChat (v2.1.2; Jin Lab, Wuhan University, Wuhan, China) [51]. Global network topology and interaction strength were compared across PSC, ICC-Adjacent, and ICC-Tumor groups. A focused analysis was performed on the shift in ligand–receptor pair usage by cholangiocytes during disease progression.

To quantitatively evaluate the hyper-biosynthetic and bioenergetic phenotype, module scores for representative biosynthetic, bioenergetic, and MYC-associated transcriptional programs were calculated in cholangiocytes using the AddModuleScore function in Seurat. Pairwise group differences among PSC, ICC-adjacent, and ICC-tumor cholangiocytes were assessed using the Wilcoxon rank-sum test with Benjamini–Hochberg correction.

### 4.7. Construction and Validation of the Diagnostic Signature

Twenty-eight high-confidence genes, identified from the intersection of pseudotime lineage drivers and tumor-specific meta-programs (MP5), were mapped to the STRING database (v12.5; STRING Consortium; confidence score > 0.4) [52] to construct a Protein–Protein Interaction (PPI) network. Topological centrality was calculated using the CytoHubba plugin (v0.1; Cytoscape App Store) within Cytoscape software (v3.10.4; Cytoscape Consortium, Seattle, WA, USA) [53] via the Degree algorithm, prioritizing the five highest-degree hub genes—CDKN1A, GADD45A, PLK3, PMAIP1, and SFN—for subsequent diagnostic modeling. The corresponding degree centrality scores were 7, 4, 4, 4, and 4, respectively (Table S30).

To avoid statistical inflation caused by treating individual cells as independent observations, diagnostic modeling was performed at the sample level rather than the single-cell level. The expression values of the candidate genes were aggregated into sample-level pseudo-bulk profiles by calculating the average expression of cholangiocytes within each patient sample. This strategy reduced single-cell dropout noise and ensured that model training was based on biological samples rather than individual cells.

The primary diagnostic signature was developed using an exhaustive “best subset selection” method based on multivariate logistic regression. Utilizing the glm function (family = binomial) in R, we systematically evaluated all possible dual- and triple-gene combinations from the five-gene hub pool. The Akaike Information Criterion (AIC) served as the selection metric to optimize model fit while minimizing complexity. The diagnostic Risk Score (RS) was calculated as follows:

*RS* = *β*_0_ + *β*_1_ × Exp*_PMAIP1_* + *β*_2_ × Exp*_GADD45A_*

To benchmark the model’s performance, a comparative signature was constructed using Least Absolute Shrinkage and Selection Operator (LASSO) [54] logistic regression via the glmnet R package (v4.1.8; Stanford University, Stanford, CA, USA). The optimal penalty parameter (λ) was determined through Leave-One-Out Cross-Validation (LOOCV) to minimize binomial deviance.

Model stability and generalizability were assessed using LOOCV in the discovery cohort and an independent external validation dataset (GSE107943). Performance was quantified through Receiver Operating Characteristic (ROC) [55] curve analysis using the pROC R package (v1.18.5; CRAN, R Foundation for Statistical Computing, Vienna, Austria), with the Area Under the Curve (AUC) serving as the evaluation metric and an effectiveness threshold set at AUC > 0.7. To reduce overfitting risk in the small discovery cohort, feature selection was restricted to biologically prioritized hub genes, model complexity was controlled by AIC, and model performance was evaluated using both internal cross-validation and external validation.

### 4.8. Statistical Analysis

All statistical analyses were performed using R software (v4.4.1). Comparisons of continuous variables, including gene expression levels and pathway scores, between two groups were conducted using the Wilcoxon rank-sum test. *p* values were adjusted for multiple testing using the Benjamini–Hochberg method. Similarity between transcriptional programs in the NMF analysis was evaluated using the Jaccard similarity coefficient.

The diagnostic performance of the PMAIP1/GADD45A signature was evaluated using receiver operating characteristic (ROC) curve analysis, with the area under the curve (AUC) used as the primary performance metric. The 95% confidence intervals of AUC values were calculated using the DeLong method. Sensitivity, specificity, and accuracy were calculated at the optimal cutoff determined by the Youden index, and the results are summarized in Table S31.

For the focused 10% mitochondrial cutoff sensitivity analysis, PMAIP1 and GADD45A expression levels, as well as the mean PMAIP1/GADD45A dual-gene expression score, were compared between CopyKAT-defined malignant-like and non-malignant ICC-tumor cholangiocytes using the Wilcoxon rank-sum test. *p* values reported as 0 due to numerical underflow in R were presented as *p* < 2.2 × 10^−16^. Unless otherwise stated, a two-sided *p* < 0.05 was considered statistically significant.

## Data Availability

The data presented in this study are openly available in GEO (https://www.ncbi.nlm.nih.gov/geo/, accessed on 6 March 2026) repositories.

## Supplementary Materials

The following supporting information can be downloaded at: https://www.mdpi.com/article/10.3390/ijms27114826/s1.

## Abbreviations

The following abbreviations are used in this manuscript:

PSC	Primary sclerosing cholangitis
ICC	intrahepatic cholangiocarcinoma
scRNA-seq	Single-cell RNA sequencing
ICC-Adj	Intrahepatic Cholangiocarcinoma-Adjacent Non-tumor Tissue
NMF	Non-negative Matrix Factorization
PPI	Protein-protein interaction
UMAP	Uniform Manifold Approximation and Projection
CNV	copy number variation
BEAM	Branched Expression Analysis Modeling
TFs	transcription factors
MPs	meta-programs
DEGs	differentially expressed genes
ORA	Over-Representation Analysis
GO	Gene Ontology
KEGG	Kyoto Encyclopedia of Genes and Genomes
AIC	Akaike Information Criterion
RS	Risk Score
LOOCV	Leave-One-Out Cross-Validation
ROC	Receiver Operating Characteristic
AUC	Area Under the Curve
UPR	unfolded protein response
ER	endoplasmic reticulum
