# Supplementary material for: A Dual-Gene Signature of PMAIP1 and GADD45A for Early Detection of Intrahepatic Cholangiocarcinoma in the Context of Primary Sclerosing Cholangitis
Source: Int J Mol Sci. 2026 May 27;27(11):4826. doi: 10.3390/ijms27114826 (PMC13256877; doi:10.3390/ijms27114826)
Supplement: Supplementary file 1 [file ijms-27-04826-s001.zip › Fig.S1.pdf]

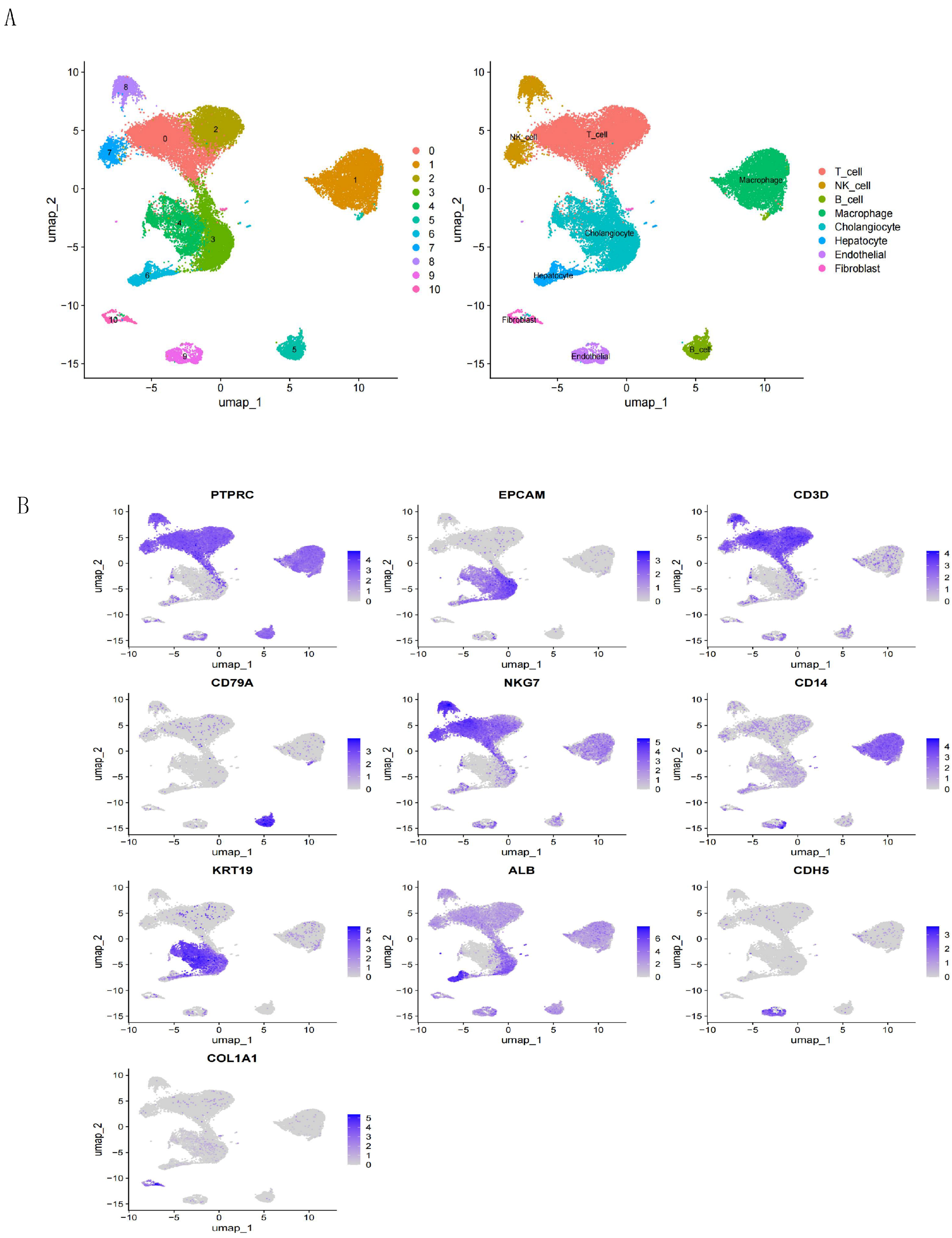

Cell clustering and marker gene expression distribution based on integrated single-cell RNA sequencing data. (A) Results of UMAP dimensionality reduction and clustering analysis of integrated single-cell RNA sequencing data, identifying 11 cell clusters and displaying the corresponding cell type distribution characteristics of each cluster.
(B) Expression distribution of key cell type marker genes (PTPRC, EPCAM, CD3D, CD79A, NKG7, CD14, KRT19, CDH5, COL1A1, etc.) in the UMAP space.
