# Supplementary material for: A Dual-Gene Signature of PMAIP1 and GADD45A for Early Detection of Intrahepatic Cholangiocarcinoma in the Context of Primary Sclerosing Cholangitis
Source: Int J Mol Sci. 2026 May 27;27(11):4826. doi: 10.3390/ijms27114826 (PMC13256877; doi:10.3390/ijms27114826)
Supplement: Supplementary file 1 [file ijms-27-04826-s001.zip › Fig.S12.pdf]

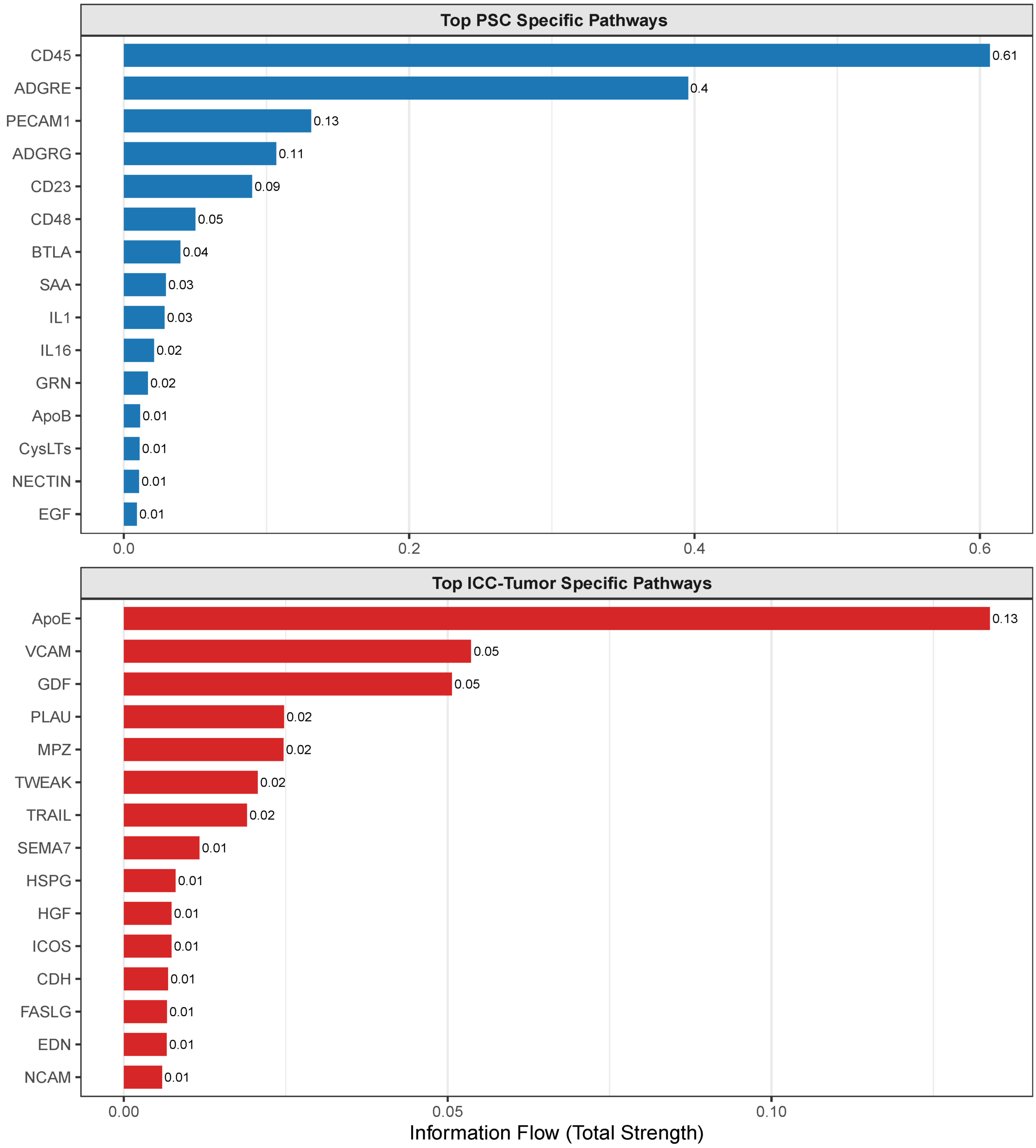

Ranking of Specific Cell-Cell Communication Pathways in Primary Sclerosing Cholangitis and Intrahepatic Cholangiocarcinoma Tumor Tissues Based on Communication Strength.
