# Supplementary material for: A Dual-Gene Signature of PMAIP1 and GADD45A for Early Detection of Intrahepatic Cholangiocarcinoma in the Context of Primary Sclerosing Cholangitis
Source: Int J Mol Sci. 2026 May 27;27(11):4826. doi: 10.3390/ijms27114826 (PMC13256877; doi:10.3390/ijms27114826)
Supplement: Supplementary file 1 [file ijms-27-04826-s001.zip › Fig.S20.pdf]

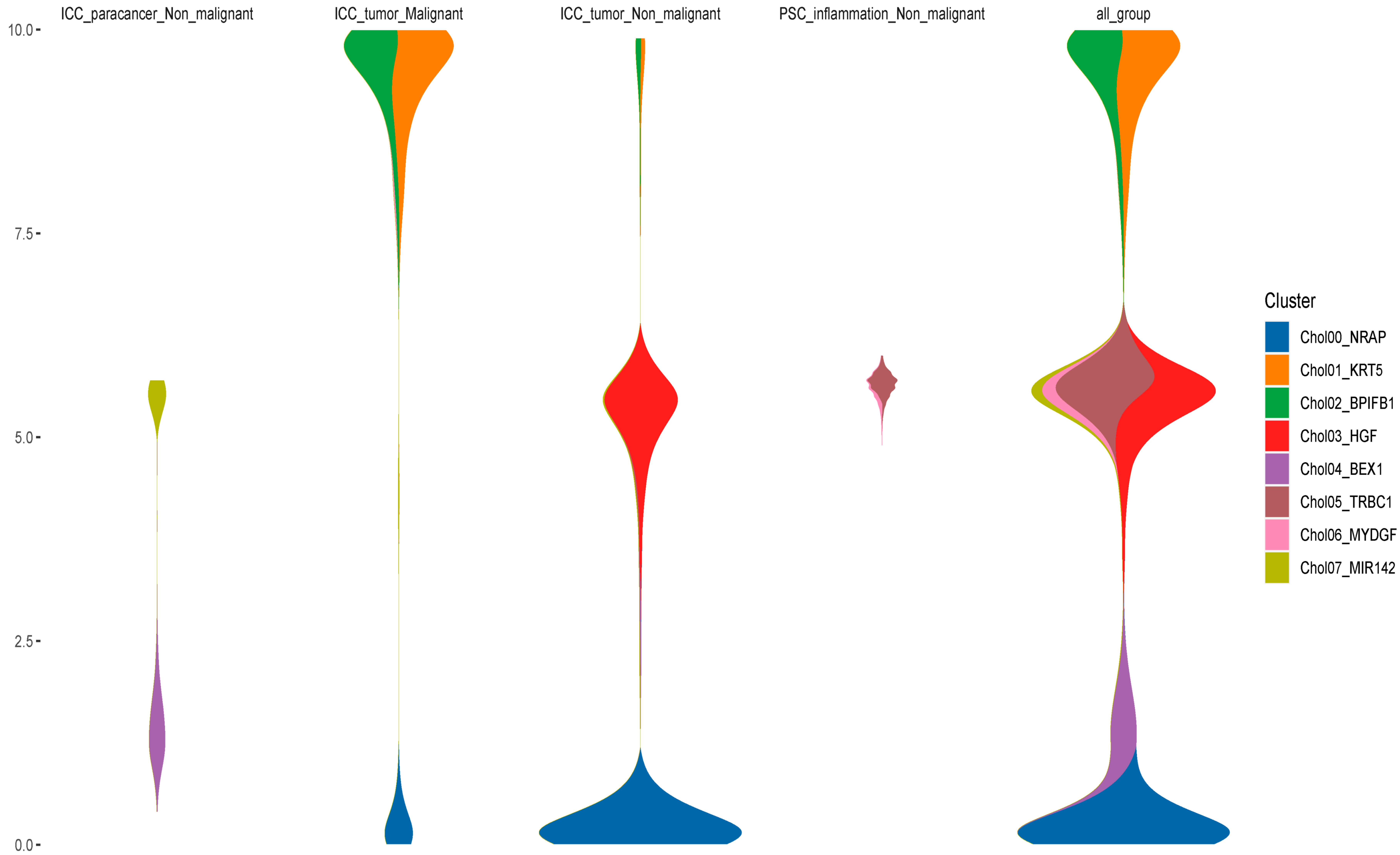

Proportion Distribution of Cell Types in Different Sample Groups During Pseudotime Progression.
