# Supplementary material for: A Dual-Gene Signature of PMAIP1 and GADD45A for Early Detection of Intrahepatic Cholangiocarcinoma in the Context of Primary Sclerosing Cholangitis
Source: Int J Mol Sci. 2026 May 27;27(11):4826. doi: 10.3390/ijms27114826 (PMC13256877; doi:10.3390/ijms27114826)
Supplement: Supplementary file 1 [file ijms-27-04826-s001.zip › Fig.S22.pdf]

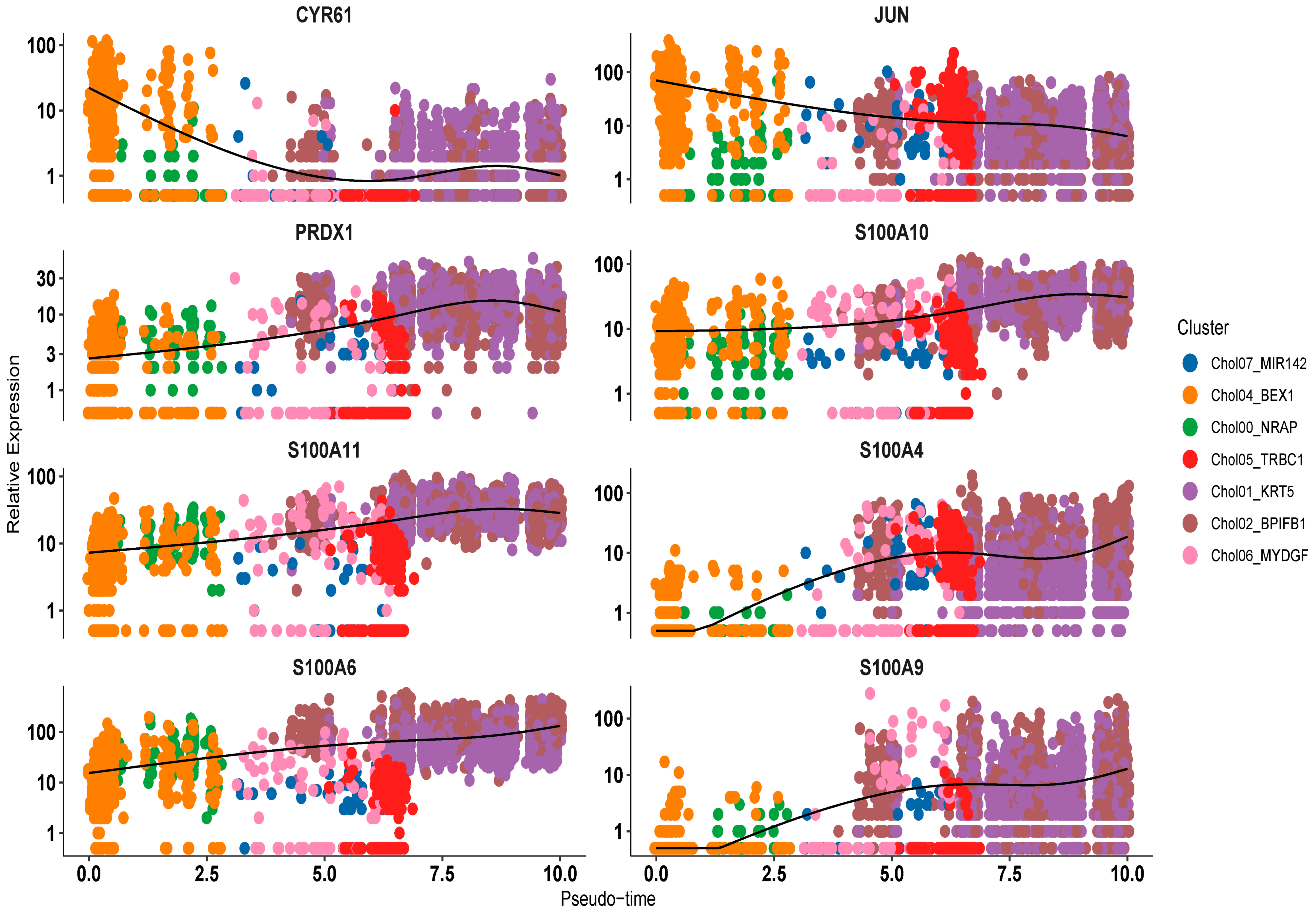

Expression Dynamics Analysis of Differentially Expressed Genes During Pseudotime Progression. Displays the expression trends of the top 8 differentially expressed genes sorted in reverse order by q-value as pseudotime progresses, with the horizontal axis representing the pseudotime process from small to large, the vertical axis representing gene expression levels, and different colors representing different cell types, which intuitively presents the temporal specificity characteristics of gene expression.
