# Supplementary material for: A Dual-Gene Signature of PMAIP1 and GADD45A for Early Detection of Intrahepatic Cholangiocarcinoma in the Context of Primary Sclerosing Cholangitis
Source: Int J Mol Sci. 2026 May 27;27(11):4826. doi: 10.3390/ijms27114826 (PMC13256877; doi:10.3390/ijms27114826)
Supplement: Supplementary file 1 [file ijms-27-04826-s001.zip › Fig.S25.pdf]

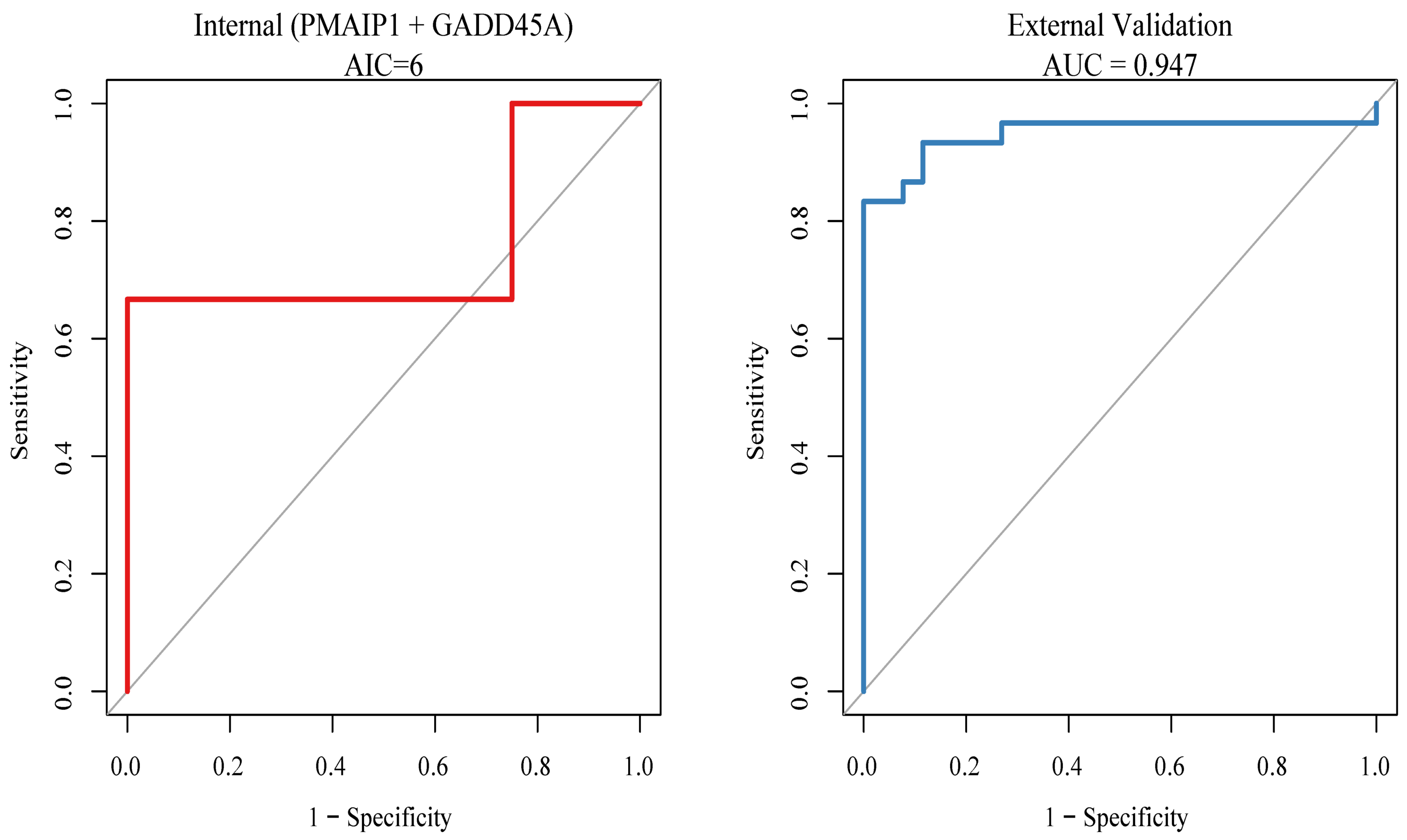

Efficacy Validation of the Indicator Combination (PMAIP1+GADD45A) Based on All-subset Selection Method.Displays the internal evaluation and external validation results of the indicator combination (PMAIP1+GADD45A) screened by the all-subset selection method. The Akaike Information Criterion (AIC) of internal evaluation is 6, and the Area Under the Curve (AUC) of external validation reaches 0.947, which intuitively presents the evaluation efficacy of this indicator combination.
