# Supplementary material for: A Dual-Gene Signature of PMAIP1 and GADD45A for Early Detection of Intrahepatic Cholangiocarcinoma in the Context of Primary Sclerosing Cholangitis
Source: Int J Mol Sci. 2026 May 27;27(11):4826. doi: 10.3390/ijms27114826 (PMC13256877; doi:10.3390/ijms27114826)
Supplement: Supplementary file 1 [file ijms-27-04826-s001.zip › Fig.S3.pdf]

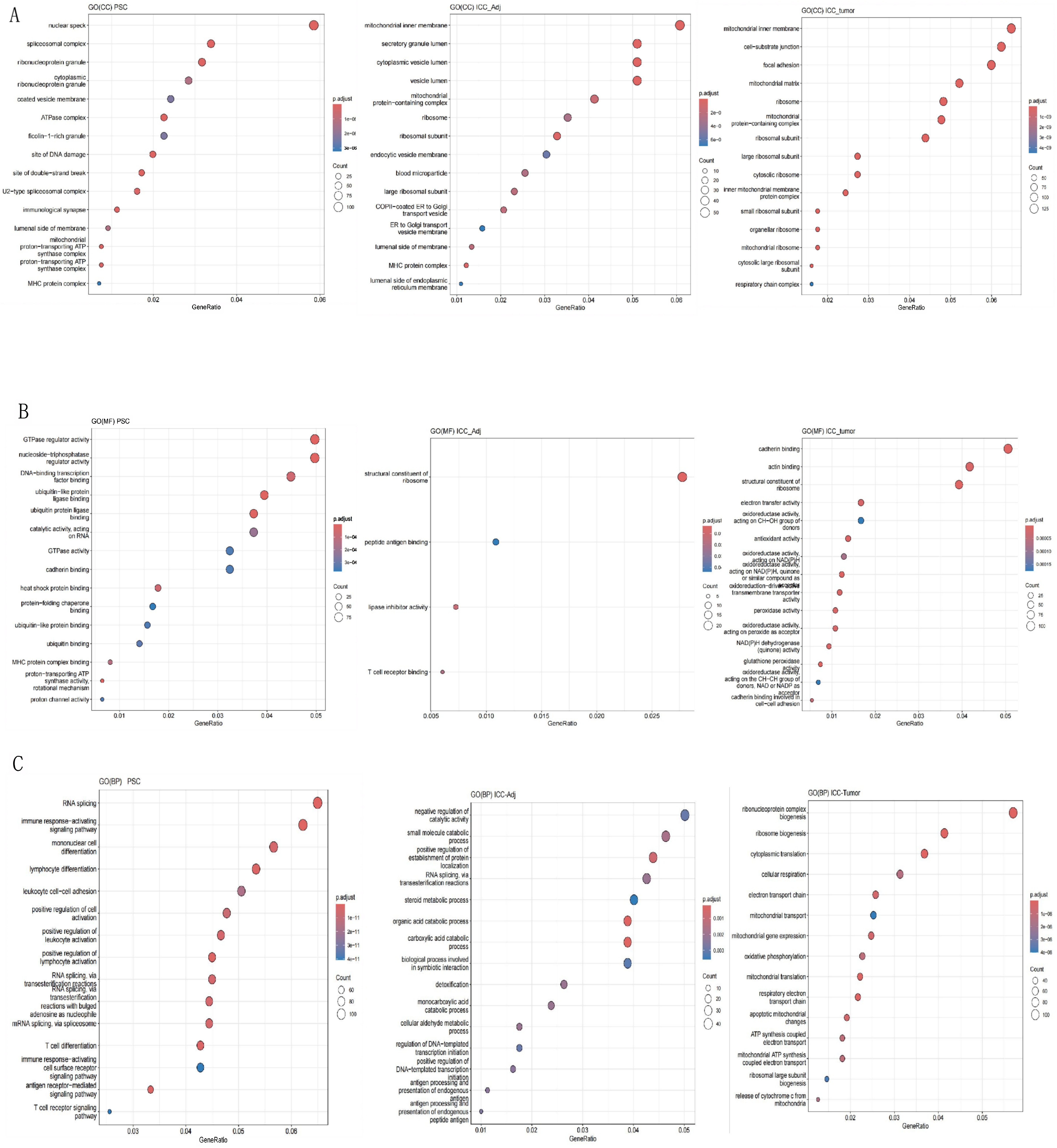

GO enrichment analysis of cholangiocyte-specific biological processes.

(A) Enrichment of Cellular Components (CC) in cholangiocytes across primary sclerosing cholangitis (PSC), intrahepatic cholangiocarcinoma adjacent non-tumor tissue (ICC-Adj), and intrahepatic cholangiocarcinoma tumor tissue (ICC-Tumor).(B) Enrichment of Molecular Functions (MF) in cholangiocytes, highlighting the key functional pathways active in different disease stages.(C) Enrichment of Biological Processes (BP) in cholangiocytes across PSC, ICC-Adj, and ICC-Tumor tissues.
