# Supplementary figures and images for: A Dual-Gene Signature of PMAIP1 and GADD45A for Early Detection of Intrahepatic Cholangiocarcinoma in the Context of Primary Sclerosing Cholangitis
Source: Int J Mol Sci. 2026 May 27;27(11):4826. doi: 10.3390/ijms27114826 (PMC13256877; doi:10.3390/ijms27114826)

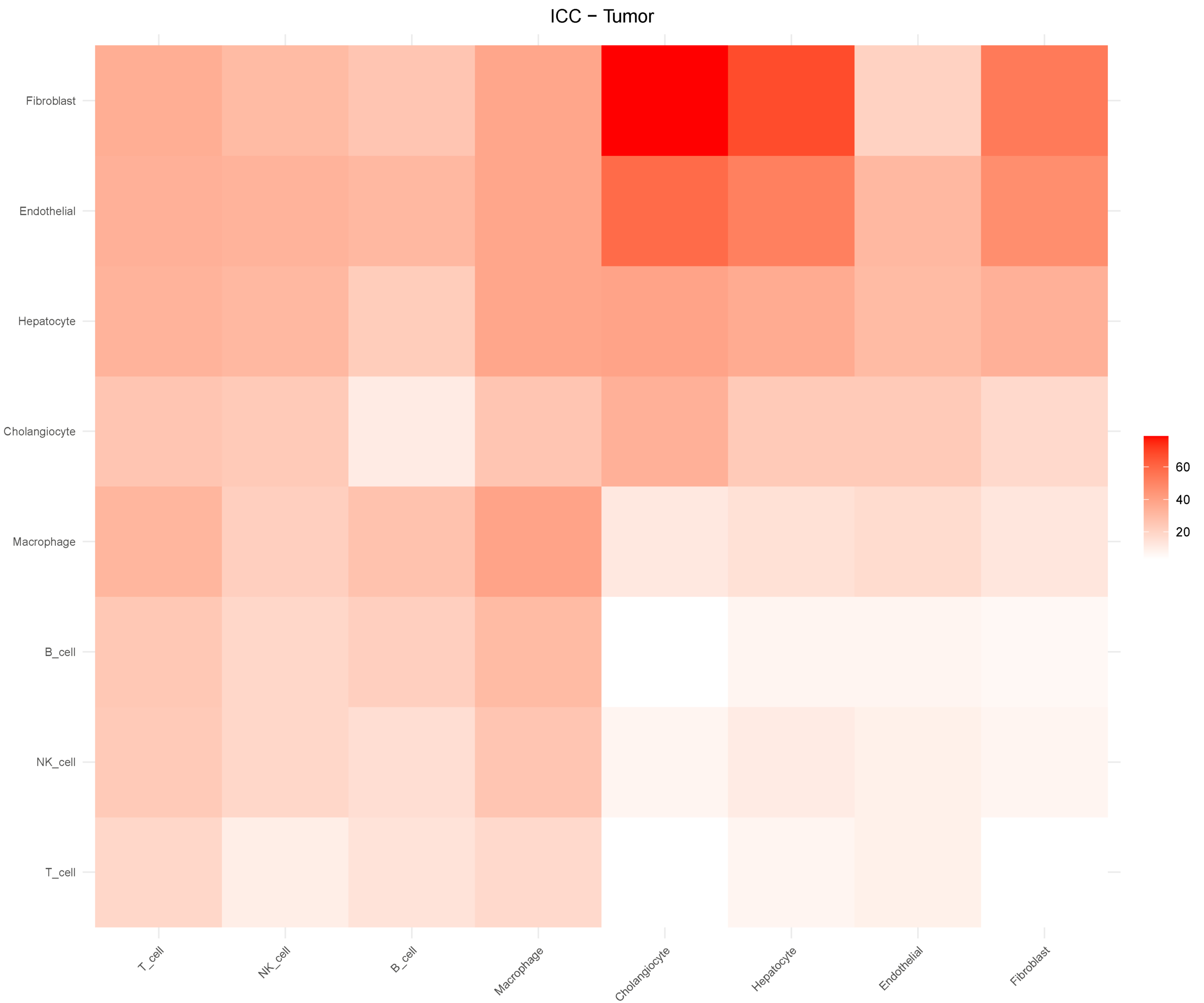

Cell-cell communication heatmap of different cell types in ICC-Tumor.

Supplement: Supplementary file 1 [file ijms-27-04826-s001.zip › Fig.S10.pdf]

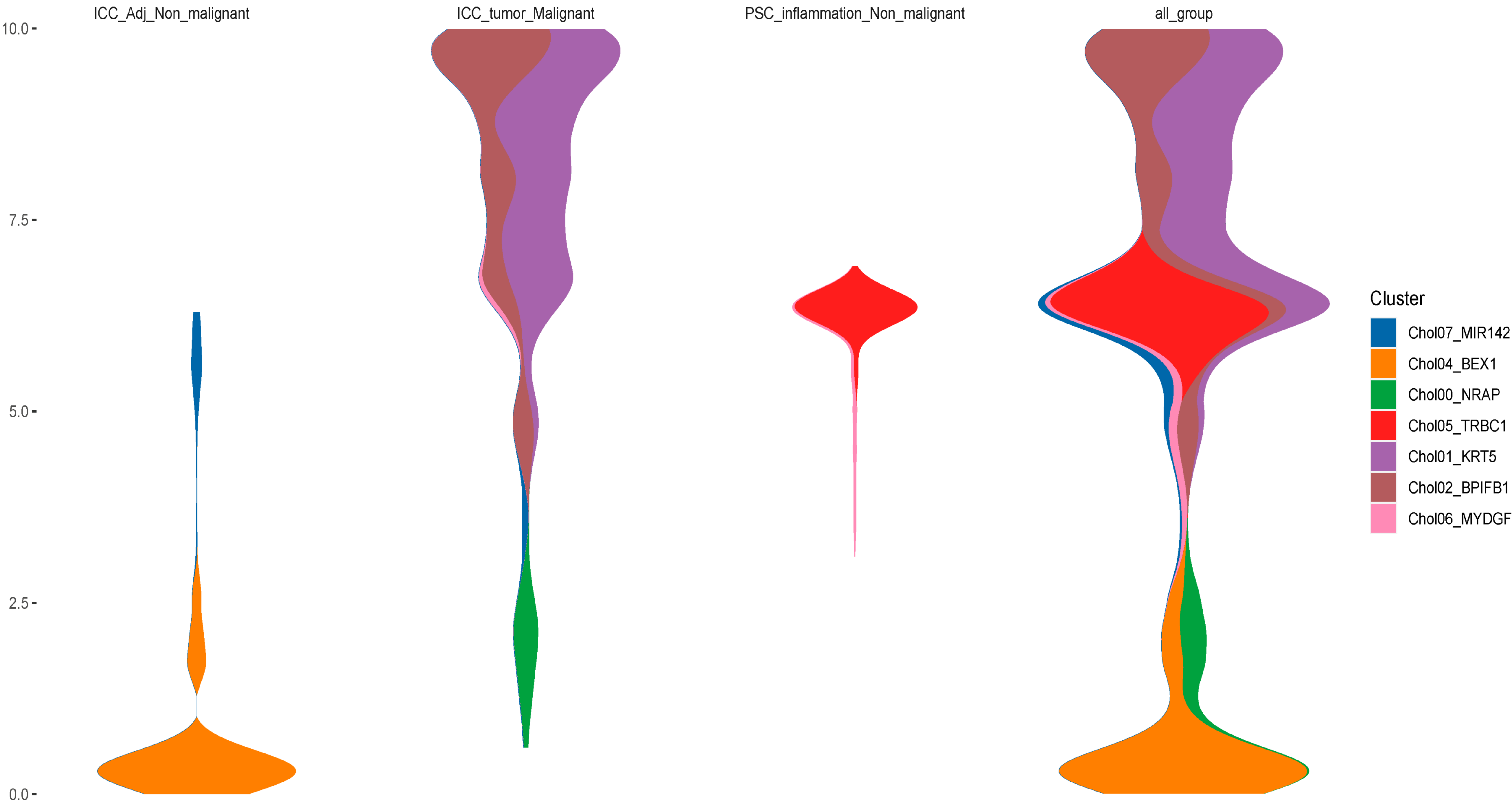

Proportion Distribution of Different Cell Types During Pseudotime Progression.

Supplement: Supplementary file 1 [file ijms-27-04826-s001.zip › Fig.S17.pdf]

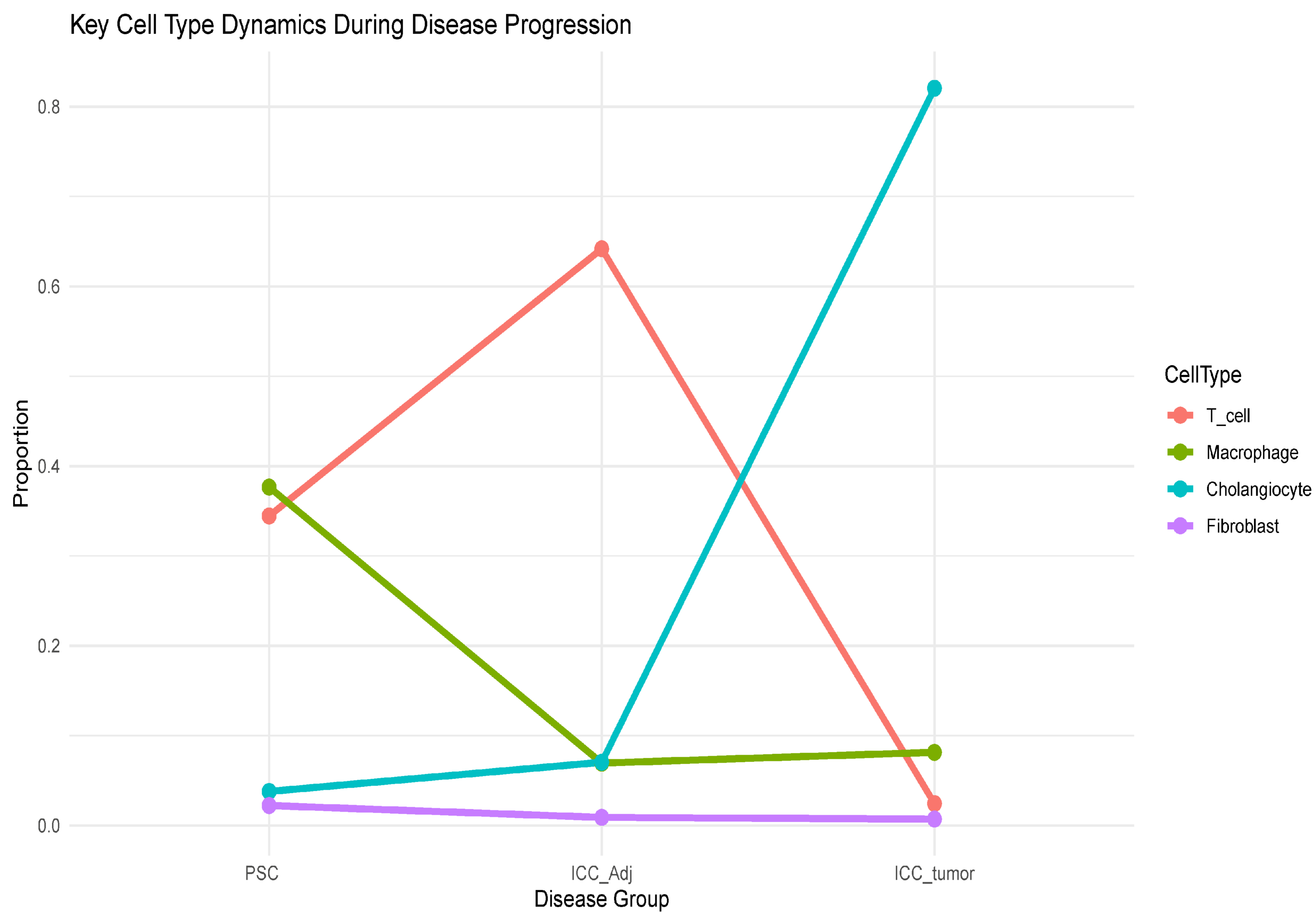

Proportion dynamics of key cell types during disease progression.

Supplement: Supplementary file 1 [file ijms-27-04826-s001.zip › Fig.S2.pdf]

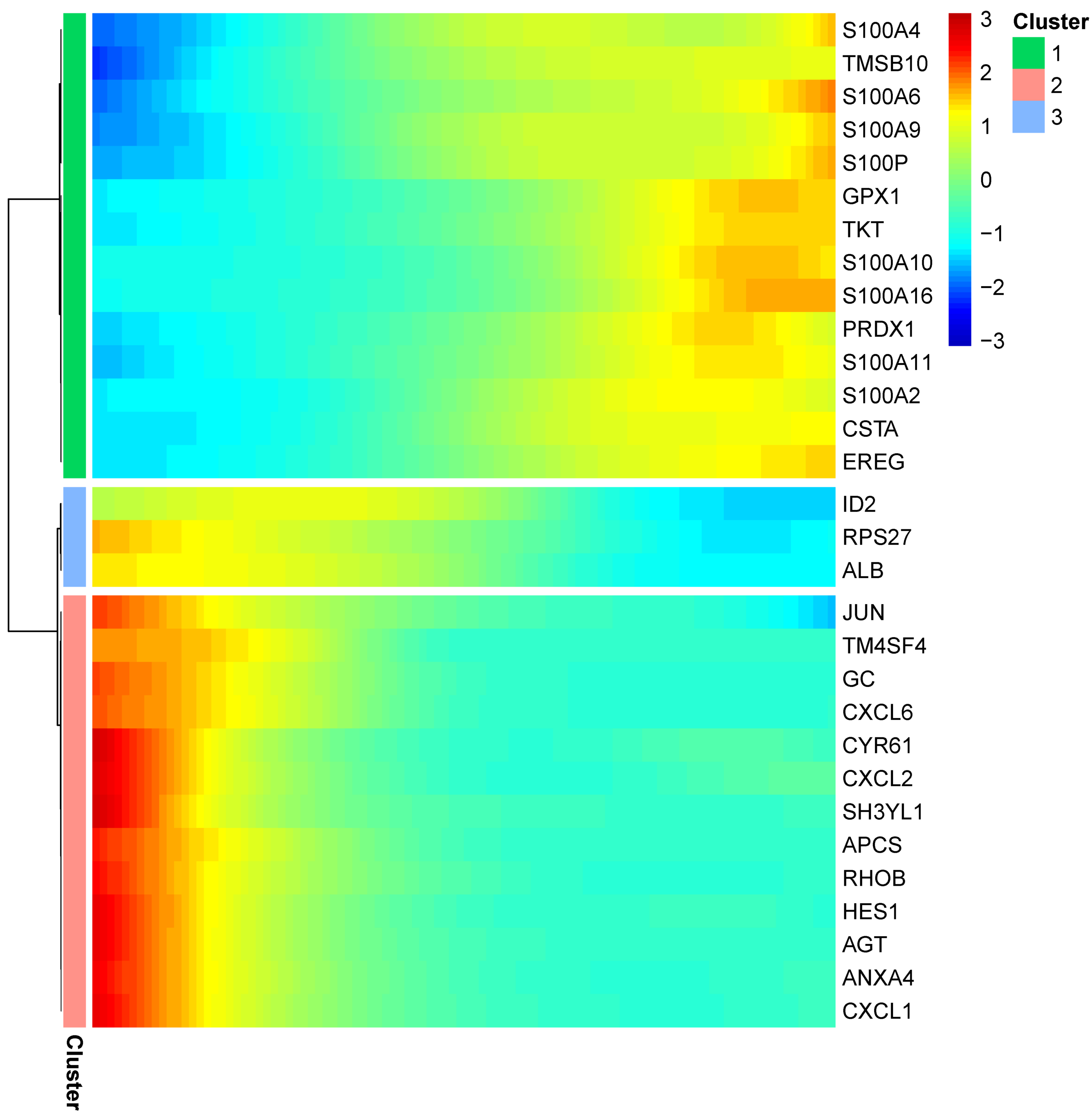

Dynamic Changes and Clustering Analysis of Gene Expression During Pseudotime Progression.

Supplement: Supplementary file 1 [file ijms-27-04826-s001.zip › Fig.S21.pdf]

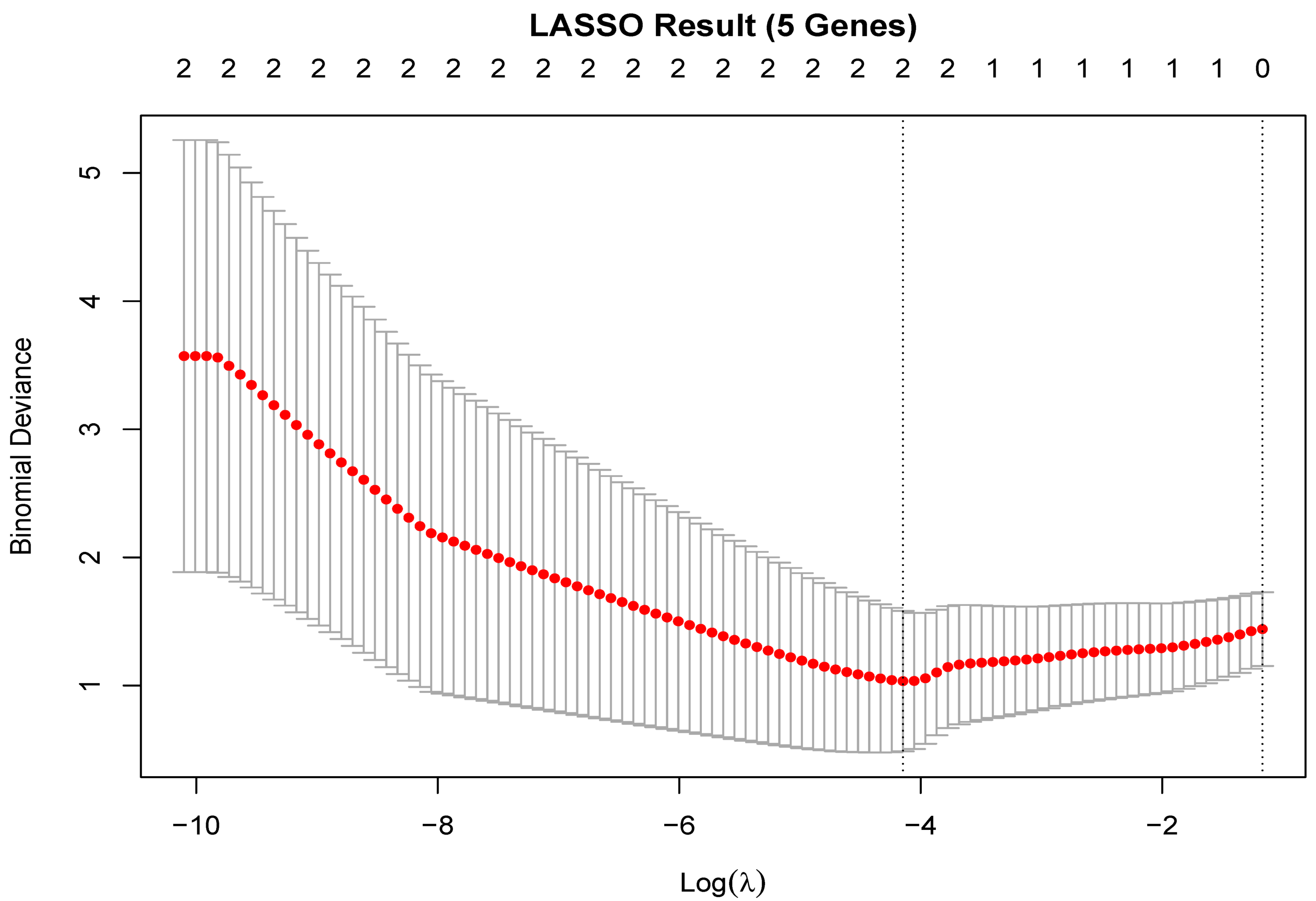

LASSO Regression Coefficient Profile Analysis Based on 5 Core Genes.

Supplement: Supplementary file 1 [file ijms-27-04826-s001.zip › Fig.S27.pdf]

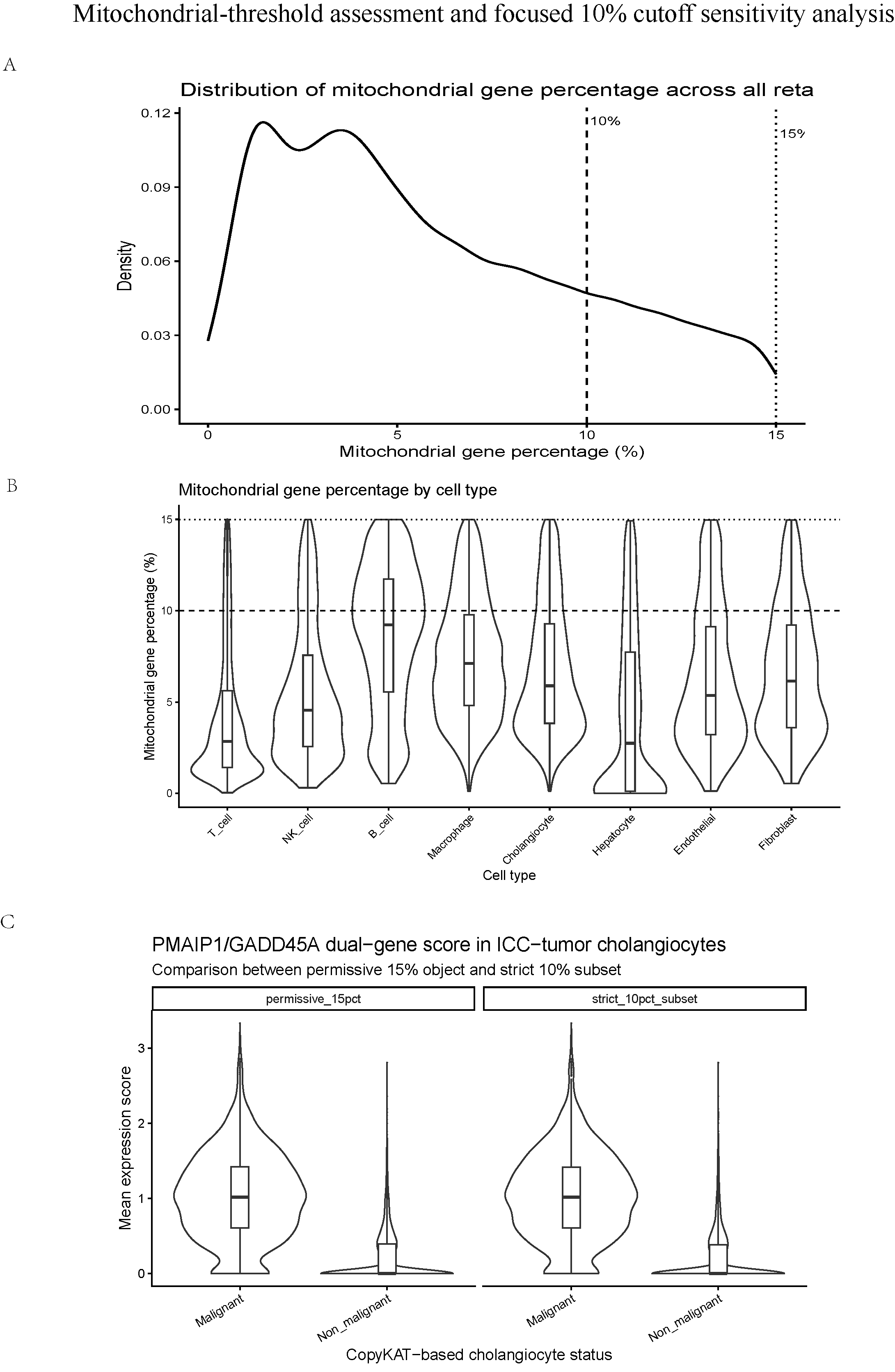

Supplement: Supplementary file 1 [file ijms-27-04826-s001.zip › Fig.S29.tif]

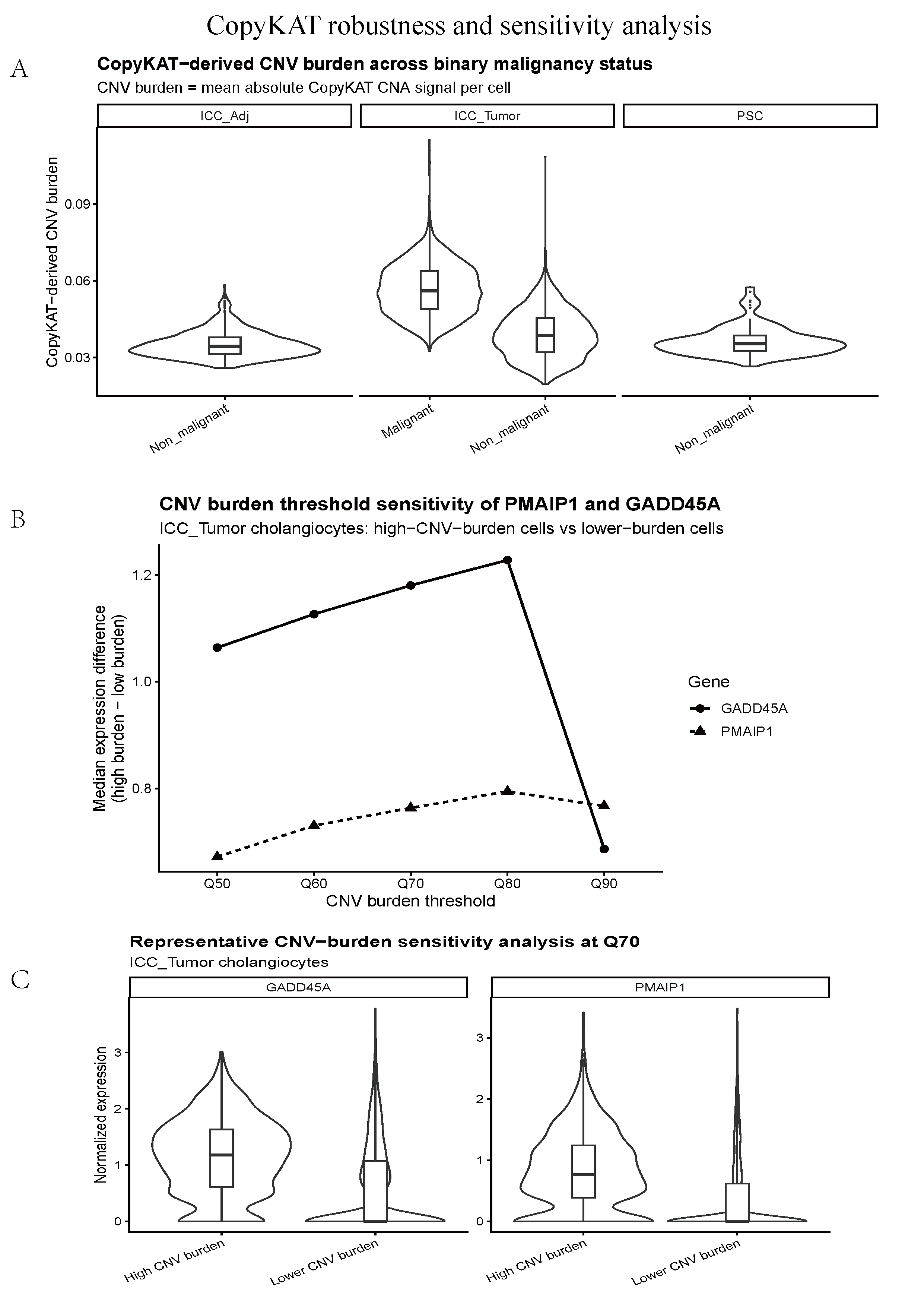

Supplement: Supplementary file 1 [file ijms-27-04826-s001.zip › Fig.S31.tif]

# Supplementary Figure : InferCNV orthogonal assessment

inferCNV

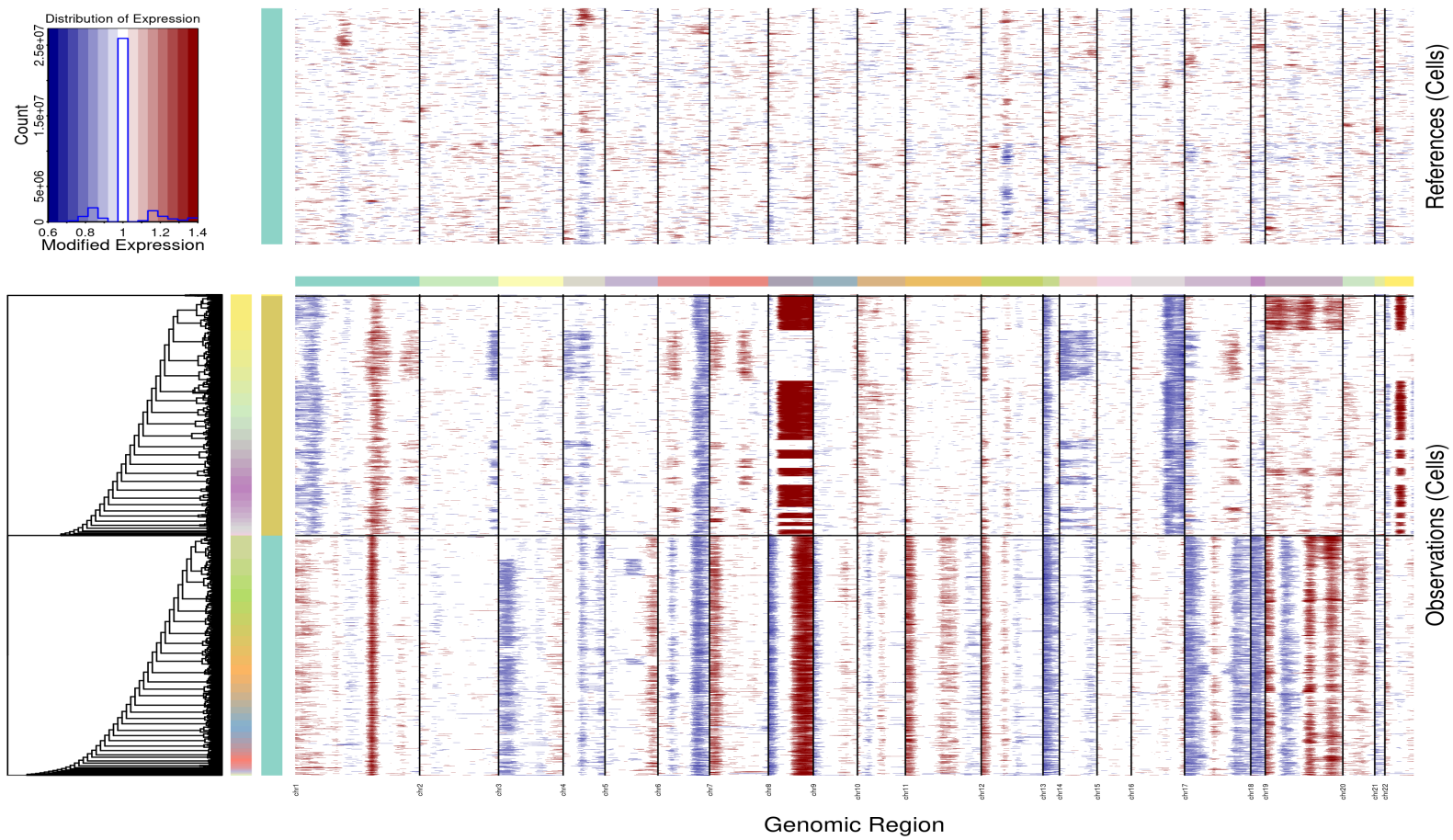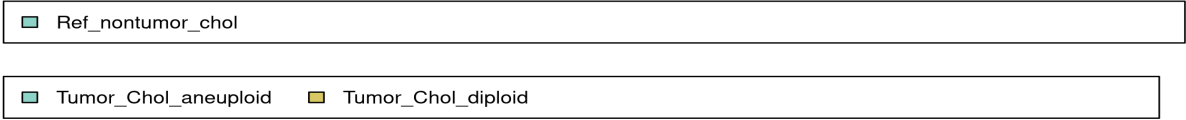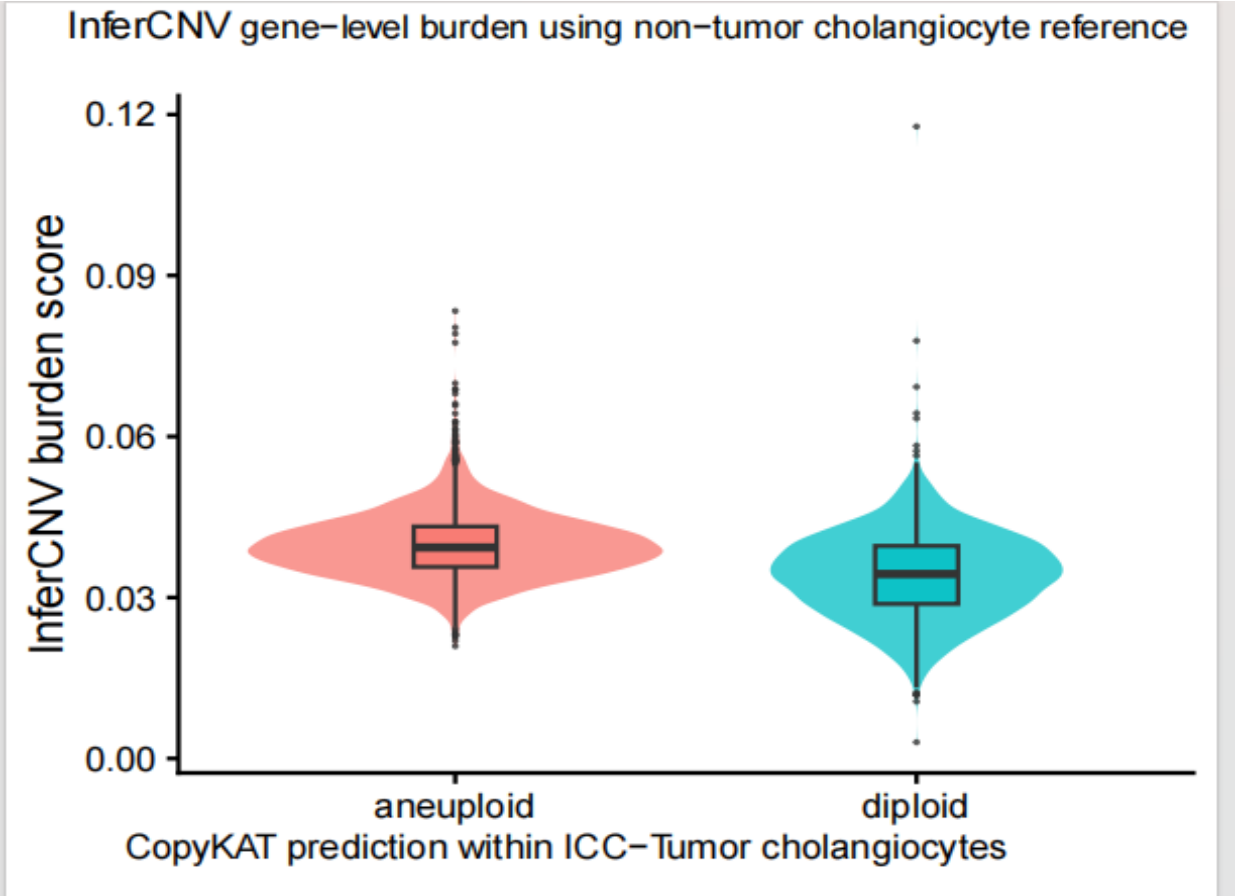

Supplement: Supplementary file 1 [file ijms-27-04826-s001.zip › Fig.S32.pdf]

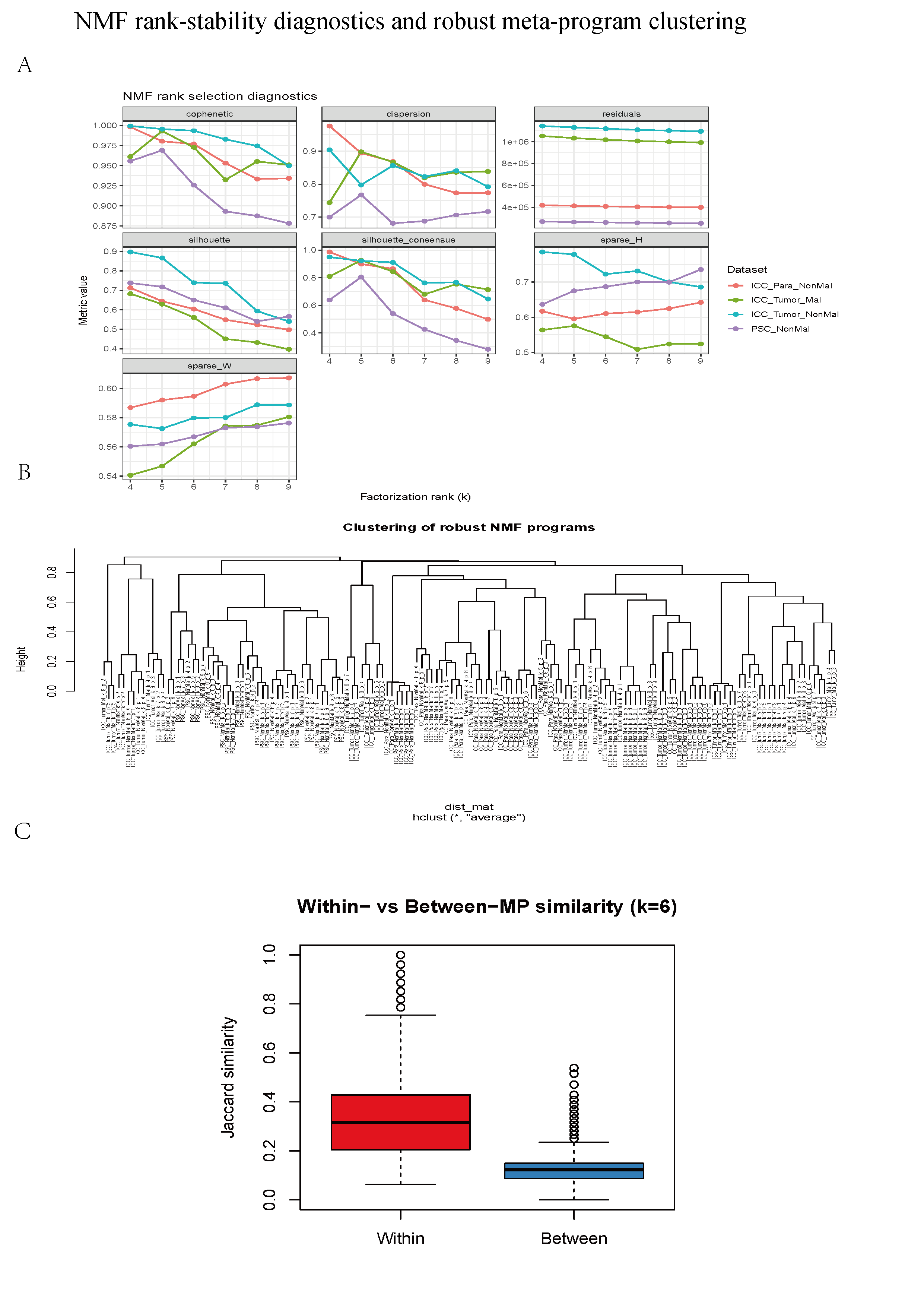

Supplement: Supplementary file 1 [file ijms-27-04826-s001.zip › Fig.S33.tif]

KEGG Enrichment – PSC Specific Genes

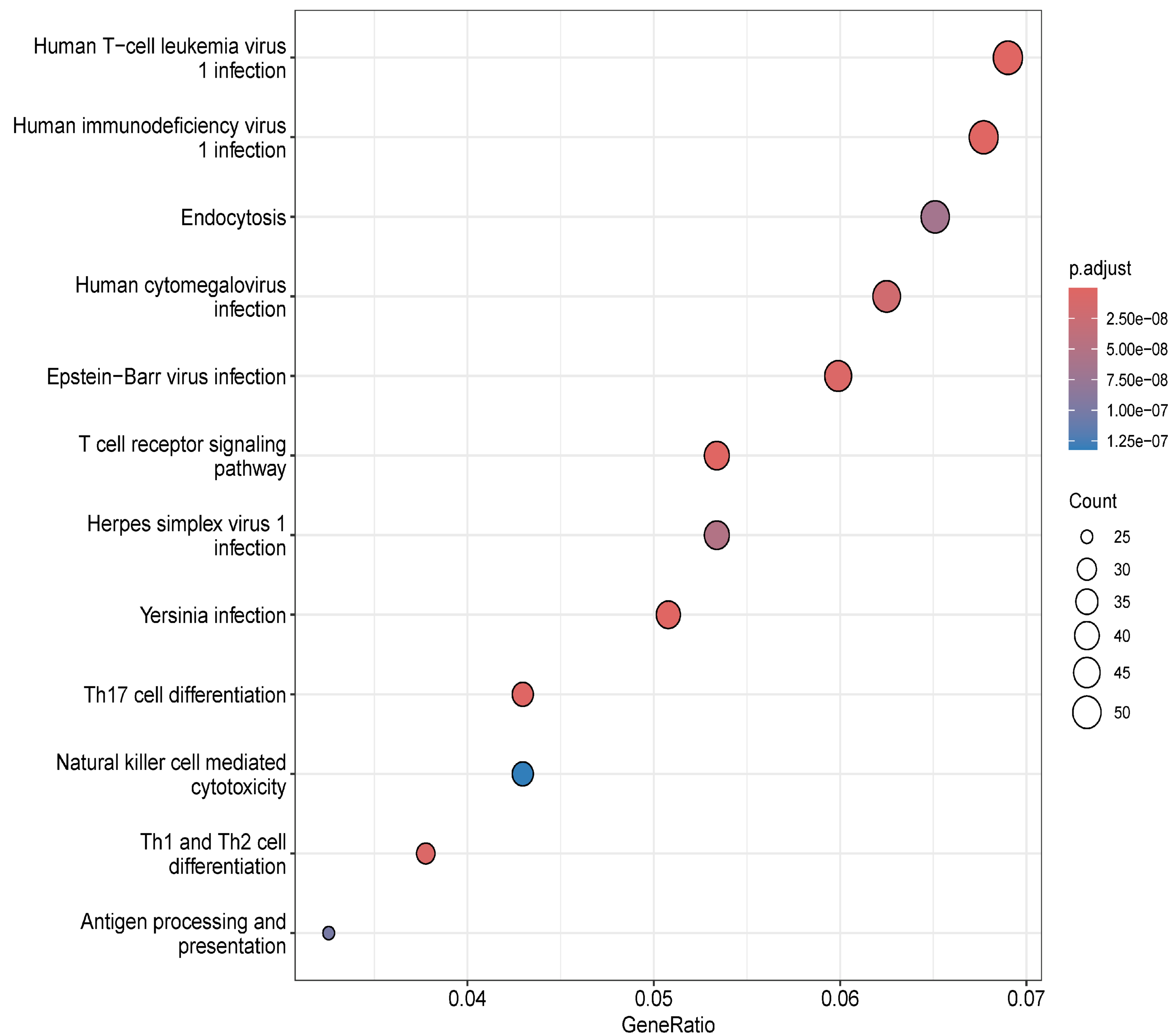

KEGG pathway enrichment analysis of PSC-specific genes.

Supplement: Supplementary file 1 [file ijms-27-04826-s001.zip › Fig.S4.pdf]

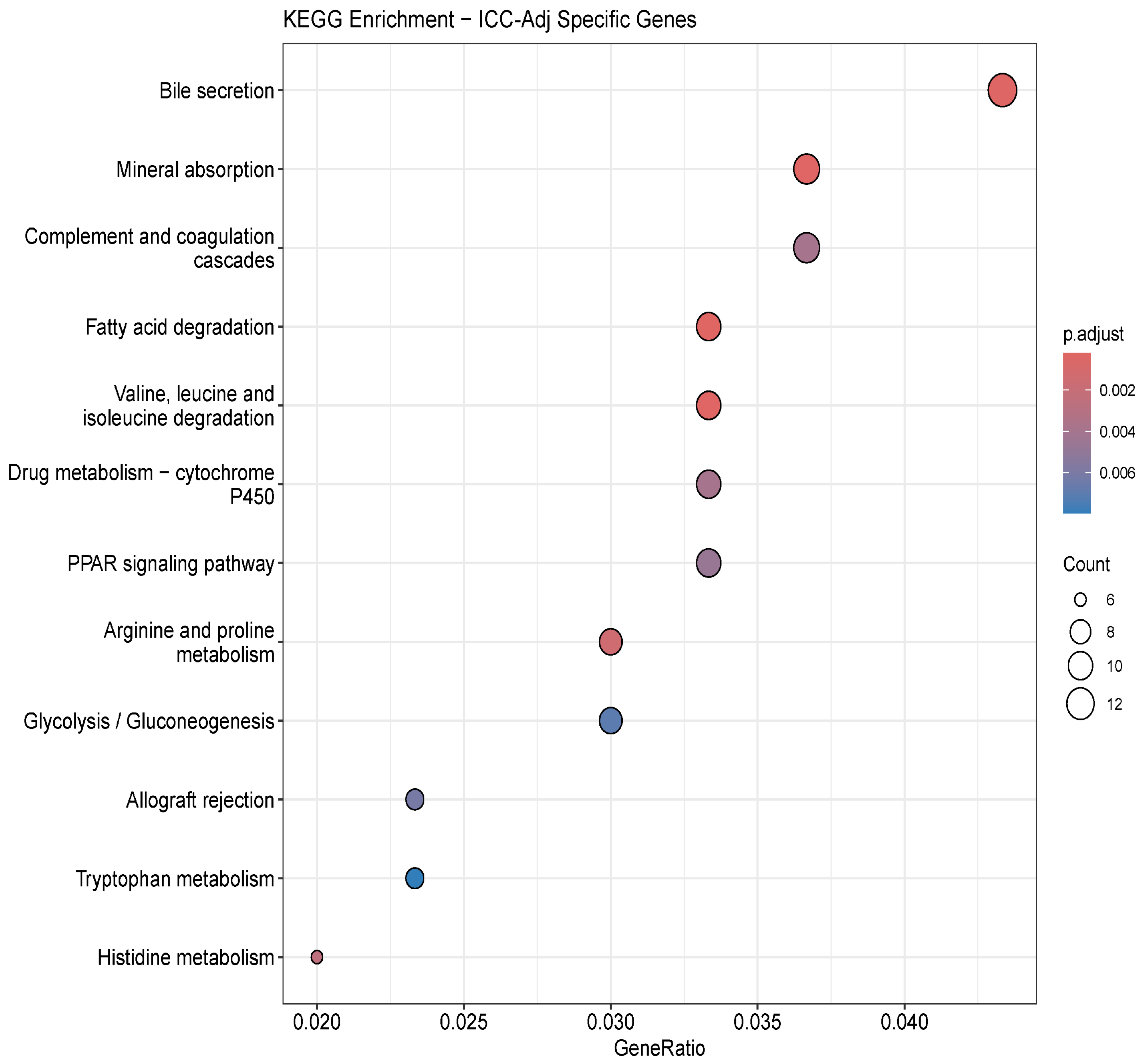

KEGG pathway enrichment analysis of ICC-Adj-specific genes.

Supplement: Supplementary file 1 [file ijms-27-04826-s001.zip › Fig.S5.pdf]

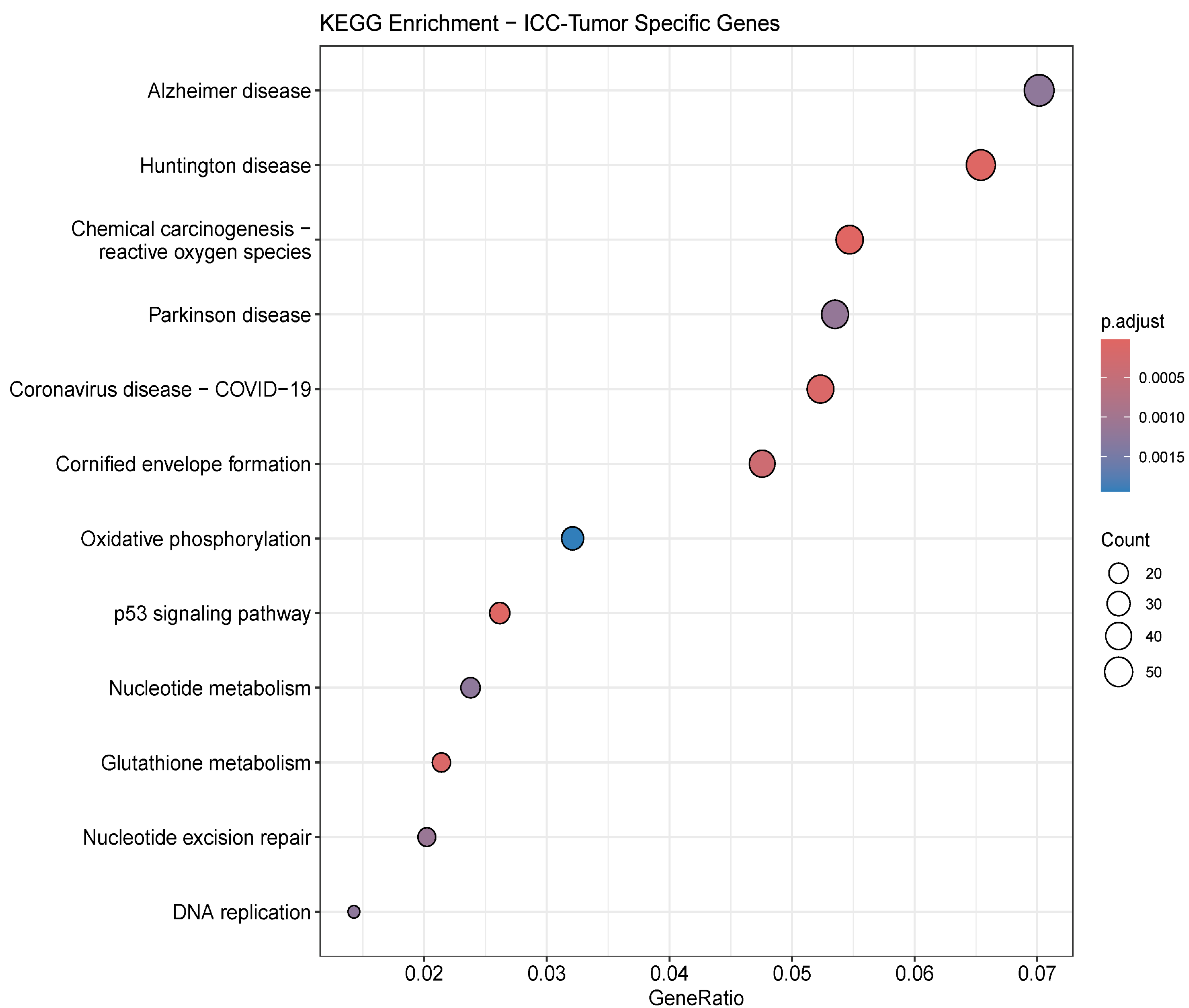

KEGG pathway enrichment analysis of ICC-Tumor-specific genes.

Supplement: Supplementary file 1 [file ijms-27-04826-s001.zip › Fig.S6.pdf]

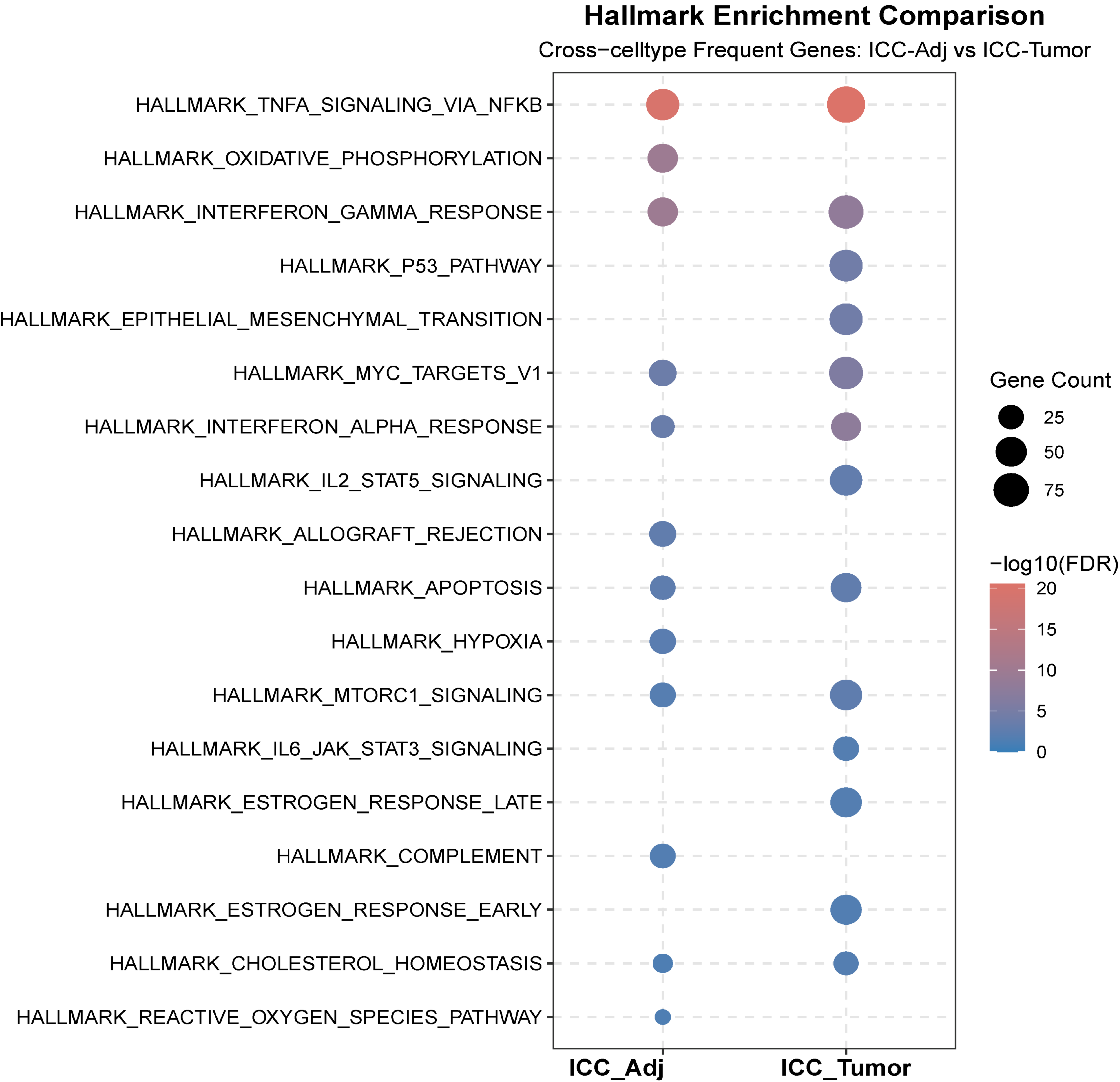

Comparative analysis of Hallmark pathway enrichment between ICC-Tumor and ICC-Adj.

Supplement: Supplementary file 1 [file ijms-27-04826-s001.zip › Fig.S7.pdf]

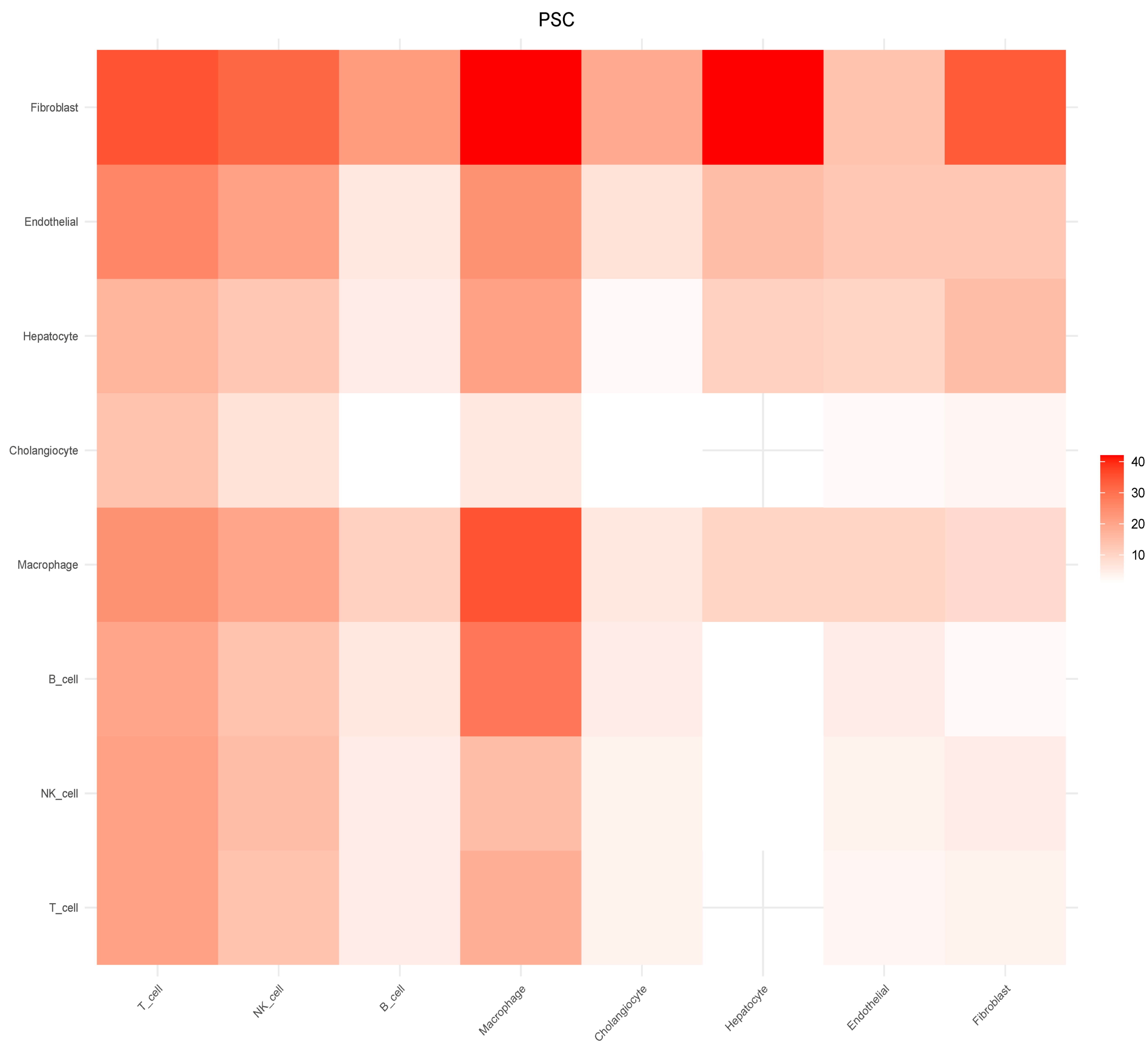

Cell-cell communication heatmap of different cell types in PSC.

Supplement: Supplementary file 1 [file ijms-27-04826-s001.zip › Fig.S8.pdf]

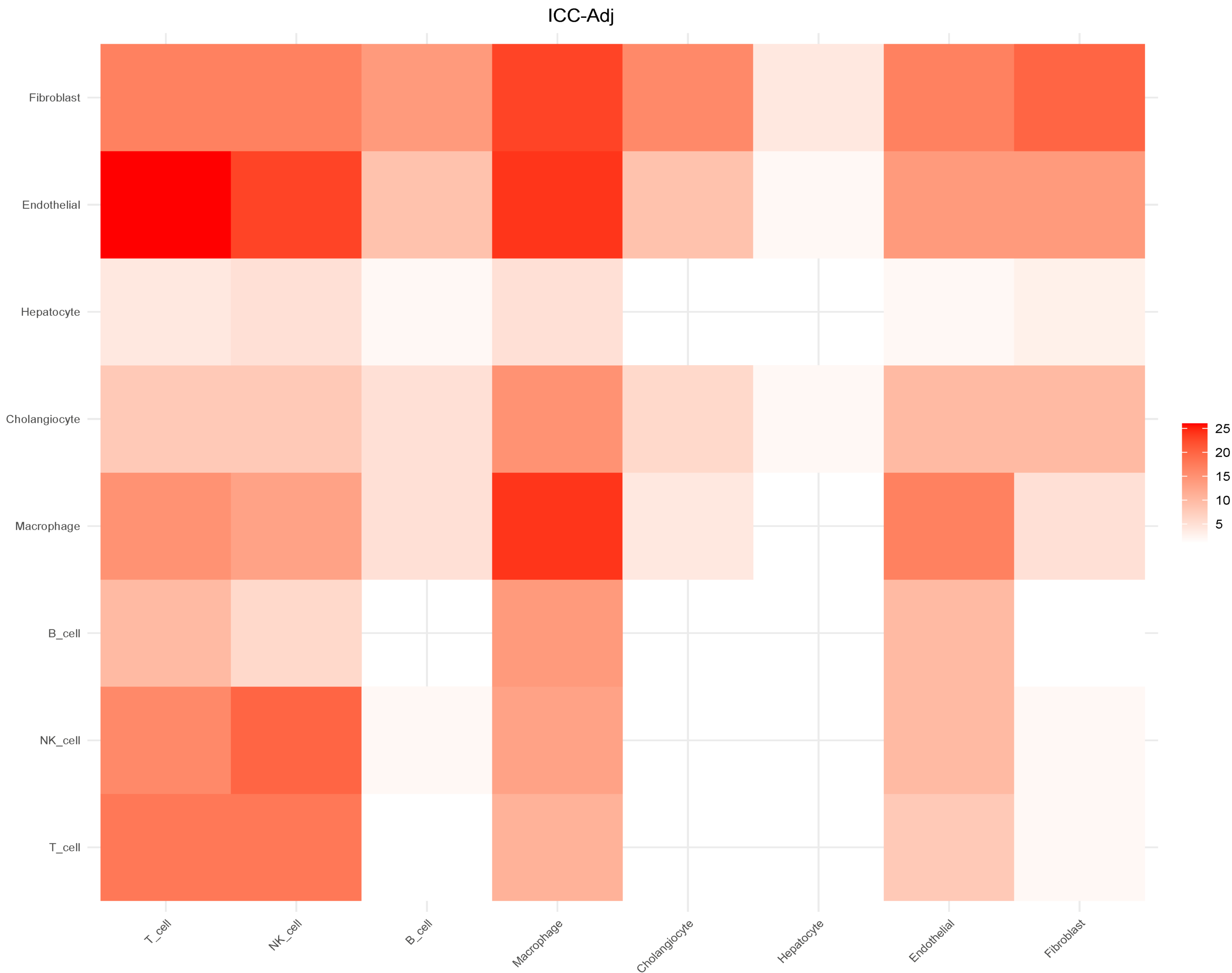

Cell-cell communication heatmap of different cell types in ICC-Adj.

Supplement: Supplementary file 1 [file ijms-27-04826-s001.zip › Fig.S9.pdf]
